# Magnetic nanoparticles for cancer theranostics

**DOI:** 10.1088/2057-1976/ae44a0

**Published:** 2026-02-19

**Authors:** Jaiden Hart, Linh Nguyen T Tran, Tamara Faranaz Ena, Niranjan A Natekar, Bahareh Rezaei, Yipeng Jiao, Hansong Zuo, Hanlei Wang, Vinit Chugh, Ebrahim Azizi, Ioannis H Karampelas, Rui He, Jenifer Gomez-Pastora, Kai Wu

**Affiliations:** 1 Department of Electrical and Computer Engineering, Texas Tech University, Lubbock, TX, United States of America; 2 Department of Chemical Engineering, Texas Tech University, Lubbock, TX, United States of America; 3 Western Digital Corporation, San Jose, CA, United States of America; 4 ASML, San Jose, CA, United States of America; 5 Lubbock High School, Lubbock, TX, United States of America; 6 Seagate Technology LLC, Shakopee, MN, United States of America; 7 Nemak USA, Inc., Sheboygan, WI, United States of America

**Keywords:** magnetic nanoparticle, cancer therapy, cancer diagnosis, magnetic imaging, magnetic biosensing

## Abstract

Magnetic nanoparticles (MNPs) have emerged as a powerful tool in cancer theranostics due to their unique size-dependent magnetic properties, surface functionalization capabilities, and responsiveness to external magnetic fields. This review outlines different types of MNPs, including those composed of pure metals, metal oxides, and metallic alloys, and highlights their size-dependent magnetic behavior, such as superparamagnetism and dynamic magnetizations. We also explore the critical role of surface modification strategies in enhancing MNPs’ biocompatibility, colloidal stability, and functional versatility for targeted biomedical applications. The applications of MNPs in cancer therapy are discussed, with a focus on magnetic hyperthermia, drug and gene delivery, and a combination of various therapies. Additionally, we examine their cancer diagnostic roles in imaging techniques such as magnetic resonance imaging (MRI) and magnetic particle imaging (MPI), and emerging magnetic biosensing technologies such as giant magnetoresistance (GMR), magnetic tunnel junction (MTJ), magnetic particle spectroscopy (MPS), and nuclear magnetic resonance (NMR)-based platforms. These advances collectively establish MNPs as key components in the future of personalized cancer diagnosis and treatment.

## 1. Introduction

Over the past decade, magnetic nanoparticles (MNPs) have garnered significant attention as multifunctional tools for biomedical applications, particularly in cancer theranostics [1–6]. Defined by their nanoscale dimensions (1–100 nm) and unique magnetic behaviors, MNPs exhibit distinct physical, chemical, and biological properties compared to their bulk counterparts. Specifically, advances in nanotechnology have enabled the controlled synthesis of MNPs with tunable size, shape, composition, and magnetic properties, along with precise surface functionalization strategies to enhance their stability, biocompatibility, and targeting specificity [5–7]. Various synthesis techniques, such as co-precipitation, thermal decomposition, hydrothermal processing, and mechanochemical methods, have evolved to reliably produce high-quality MNPs suitable for biomedical use. Concurrently, surface modification techniques, ranging from polymeric coatings and inorganic shells to biomimetic and cell membrane coatings, have been employed to improve circulation time, minimize aggregation, and enable the conjugation of therapeutic and diagnostic agents [8–10]. These developments have laid the groundwork for translating MNPs into clinical settings, with several iron oxide-based formulations reaching clinical trials and some gaining regulatory approval as MRI contrast agents.

In parallel, the biomedical applications of MNPs in cancer therapy and diagnosis have expanded considerably [1, 2, 11]. In cancer treatment, MNPs serve as key agents in magnetic hyperthermia, where localized heating enables minimally invasive tumor ablation [12–14]. Additionally, MNPs are increasingly utilized for targeted drug and gene delivery, offering site-specific accumulation, controlled release, and minimal systemic toxicity [15–18]. Combination therapies that integrate magnetic hyperthermia with chemotherapy or gene therapy are being actively investigated to enhance therapeutic efficacy. In diagnostics, MNPs have revolutionized magnetic resonance imaging (MRI) [19–23] as contrast enhancers and have enabled the development of magnetic particle imaging (MPI) [24–26], a novel imaging modality that allows for real-time, radiation-free tracking of MNP tracers. Moreover, MNP-based biosensors leveraging magnetoresistance (MR) [27–33], magnetic particle spectroscopy (MPS) [34–37], and nuclear magnetic resonance (NMR) [38–41] technologies have shown promise for highly sensitive molecular diagnostics and cancer biomarker detection.

This review aims to provide a comprehensive overview of MNPs in cancer theranostics. Section 2 discusses the fundamental properties of MNPs, including their classification, synthesis, magnetic behavior, and surface functionalization strategies. Section 3 focuses on their therapeutic applications in hyperthermia, drug and gene delivery, and combinatorial treatment approaches. Section 4 reviews diagnostic applications, including MNP-enhanced MRI, MPI, and various magnetic biosensing platforms. Section 5 outlines the current challenges hindering clinical translation, such as toxicity and synthesis reproducibility, and proposes future directions to overcome these limitations. Finally, section 6 concludes with a summary of the field’s progress and emphasizes the opportunities for advancing MNP-based cancer theranostics through interdisciplinary research. Overall, this review aims to highlight recent developments, identify emerging trends, and emphasize the translational potential of MNPs in precision oncology.

## 2. MNPs: properties and functionalization

### 2.1. Types of MNPs

#### 2.1.1. Definition of MNP

**Figure 1.**
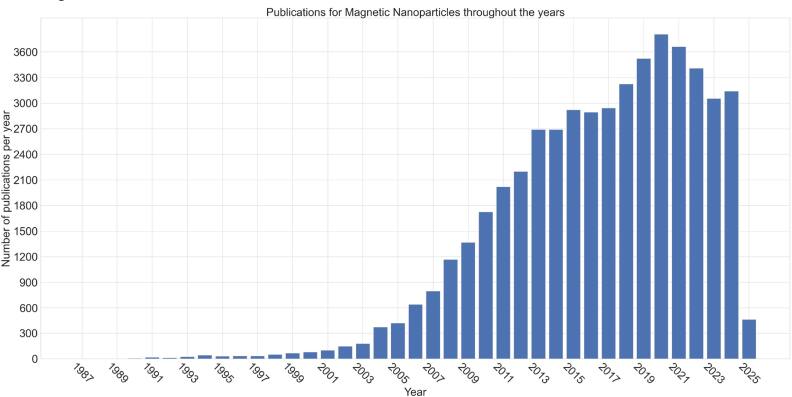
Annual number of publications related to MNPs indexed in the PubMed database between 1987 and 2025. The bar plot reflects the progressive growth of research output in this field over the past three decades. The *Y*-axis indicates the number of indexed publications per year, and the *X*-axis denotes publication year. Original figure prepared by the authors.

MNPs are nanoscale materials that exhibit magnetic behavior and can be precisely manipulated using external magnetic fields. Their unique combination of magnetic responsiveness and tunable physicochemical properties makes them highly versatile for applications spanning biomedicine, materials science, engineering, and environmental remediation. In biomedicine, surface-engineered MNPs can enhance the stability of therapeutic agents, improve the solubility of poorly water-soluble drugs, and prolong circulation time in the bloodstream, enabling more efficient and targeted delivery. Biocompatible and biodegradable types, such as iron oxide nanoparticles (IONPs), offer additional advantages, including minimal residual waste after degradation and compatibility with biological molecules and tissues. MNPs can also be functionalized with targeting ligands, imaging probes, or therapeutic cargos, allowing integration of diagnosis and therapy within a single platform. Beyond medicine, MNPs are used in materials science and engineering to enhance the performance of electronic components, and in environmental science for detecting and removing contaminants from water. Their high surface-to-volume ratio, tunable surface chemistry, and strong magnetic responsiveness enable rapid separation and recycling in these applications. Another key advantage of MNPs is the ability to tailor their composition by using pure metals, metal oxides, or metallic alloys, and to modify their surfaces with organic or inorganic coatings. These strategies expand their functional range, improve stability in diverse environments, and lower production costs, making them suitable for both high-tech and large-scale applications. Figure 1 highlights the steady increase in publications on MNPs in science and medicine, underscoring their growing potential in addressing major technological and health challenges such as cancer.

#### 2.1.2. Pure metal MNPs

For pure metal MNPs, transition elements with unpaired d-electrons exhibit enhanced magnetic properties due to their high saturation magnetization (M_s_). These characteristics make them ideal candidates for MNP applications. Among them, iron (Fe) and cobalt (Co) are particularly attractive because of their abundance, ease of synthesis, and strong magnetic performance [42].

**Figure 2.**
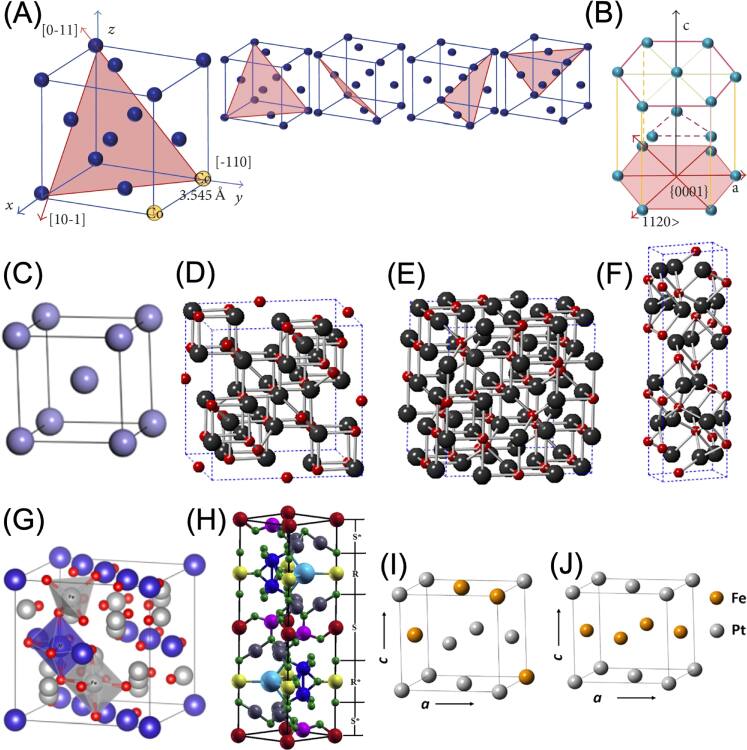
(A) FCC and (B) HCP structures of Co. The planes in red are slip planes (which indicate directions in which atoms can slip, leading to plastic deformation). (C) BCC structure of Fe. Crystal structures for (D) Fe_3_O_4_, (E) $\gamma $- Fe_2_O_3_, and (F) $\alpha $- Fe_2_O_3_. Red spheres indicate oxygen atoms, while black spheres indicate Fe atoms. (G) Inverse spinel structure for MFe_2_O_4_ type MNPs. Silver spheres represent Fe atoms, while red spheres represent oxygen atoms. The purple spheres represent metal ‘M’ atoms (M = Fe(II), Co(II), Ni(II), Mn(II)). (H) Example of a hexagonal ferrite structure of the form MFe_12_O_19_. This structure represents M-type Barium Hexaferrite. Red, pink, gray, blue, and yellow-colored spheres represent iron atoms in 2a, 4f1, 12k, 4f2, and 2b sites, respectively. S, R, S*, and R* are used to represent five levels of the structure, with the ‘*’ indicating a 180° rotated level. Small green spheres represent oxygen atoms, while large cyan spheres represent barium atoms. (I) and (J) are the representations of the crystal structure for the A1 phase (I) and L10 phase (J) FePt. (A) and (B) reprinted from [45], licensed under CC BY 4.0. (C) reprinted from [46], licensed under CC BY 4.0. (D)–(F) reprinted with permission from [47], copyright 2022 American Chemical Society. (G) reprinted with permission from [48], copyright 2020 Springer Nature Switzerland AG. (H) reprinted with permission from [49], copyright 2014 Elsevier B.V., licensed under CC BY-NC-ND 3.0. (I) and (J) reprinted with permission from [42], copyright 2023 American Chemical Society.

Cobalt exists naturally in either the hexagonal close-packed (HCP) or face-centered cubic (FCC) crystal structures (figures 2(A)&(B)), with the HCP-to-FCC transition occurring at approximately ∼417 °C. The magnetocrystalline anisotropy constant for HCP Co (440 kJ m^−3^) is higher than that of FCC Co (270 kJ m^−3^) [43]. However, both anisotropy values are lower than those of certain transition-metal alloys such as FePt, FePd, and CoPt, and much lower than rare-earth magnets such as SmCo_5_ (17,200 kJ m^−3^) and Nd_2_Fe_14_B (4,900 kJ m^−3^). Since anisotropy is also shape-dependent, certain morphologies can yield higher anisotropy values; for instance, Co nanowires have shown anisotropy values up to 1,130 kJ m^−3^ [44], significantly exceeding both HCP and FCC Co.

Iron predominantly crystallizes in the body-centered cubic (BCC) structure (figure 2(C)). Its anisotropy and coercivity are low. However, the high number of unpaired d-electrons gives Fe a high saturation magnetization (M_s_) of 221 Am^2^ kg^−1^. This combination of high M_s_ and low anisotropy classifies Fe as a soft magnetic material—well-suited for applications in magnetic transport and other high-moment uses. Fe MNPs can be produced using a range of physical methods (lithography, mechanical milling, laser evaporation, wire explosion, gas-phase synthesis) and chemical methods (precipitation/co-precipitation, sol–gel, hydrothermal, microemulsion, polyol, sonochemical synthesis). Thermal decomposition, in particular, has been shown to provide excellent control over particle size and magnetic property distribution [5].

#### 2.1.3. Metal oxide MNPs

For biomedical applications, MNPs must be biocompatible, readily available, biodegradable without harmful residues, and cost-effective to produce. IONPs fulfill these requirements and are widely used in medicine. Their performance can be enhanced through surface functionalization with bioactive molecules such as antibodies, peptides, and small molecules, enabling targeted tumor accumulation. At the nanoscale, IONPs exhibit superparamagnetism, making them highly responsive to external magnetic fields and capable of producing strong local gradients. These properties make them excellent MRI contrast agents, effective mediators of magnetic hyperthermia, and promising platforms for precision drug delivery in cancer therapy. Like other iron-based MNPs, IONPs can be synthesized via physical methods—lithography, mechanical milling, laser evaporation, wire explosion, and gas-phase synthesis—or chemical methods such as precipitation/co-precipitation, sol–gel processing, hydrothermal synthesis, microemulsion, polyol methods, thermal decomposition, and sonochemistry.

Metal oxides such as *γ*-Fe_2_O_3_ and MFe_2_O_4_ (ferrites, where M = Co, Fe, Ni, Mn) adopt a cubic ferrite structure (figures 2(D)-(F)) and share many of the advantageous magnetic properties of IONPs. Ferrite MNPs can efficiently generate heat under an alternating magnetic field (AMF), making them suitable for magnetic hyperthermia. Their superparamagnetic nature ensures zero net magnetization in the absence of a field while allowing rapid magnetic alignment when a field is applied. This behavior prevents aggregation after field removal, improving circulation time for therapeutic applications. Their small size and large surface-to-volume ratio enable effective tumor targeting, while surface coatings (e.g., polyethylene glycol, PEG) enhance biocompatibility, facilitate biodegradation, and minimize environmental impact.

The magnetic anisotropy of ferrites depends on particle shape and the balance between shape and crystalline anisotropy. In the inverse spinel structure of MFe_2_O_4_ (figure 2(G)), two Fe atoms are antiferromagnetically coupled, and the net magnetic moment is determined by the magnetic moment of the M element. Hexagonal ferrites (figure 2(H)), with the general formula MFe_12_O_19_ (six Fe_2_O_3_ units plus MO, where M = Ba or Sr), consist of cubic MO and Fe_2_O_3_ in either cubic *γ* (magnetite) or trigonal α (hematite) phases. Hexagonal ferrites have higher magnetic anisotropy than cubic ferrites due to a well-defined anisotropy axis. Cubic ferrites typically exhibit crystalline anisotropies of 13–200 kJ m^−3^, while hexagonal ferrites range from 300−500 kJ m^−3^ [50], both exceeding the values found in metallic transition elements.

#### 2.1.4. Metallic alloy MNPs

For metallic alloy MNPs, preferred compositions generally follow the form AB, where A is a transition metal with unpaired d-electrons (e.g., Fe, Co, Ni) that provides the alloy’s magnetic properties, and B is a metal with high polarizability (typically Pt or Pd). Common examples include FePt, FePd, and CoPd. At the nanoscale size, these metallic alloy MNPs exhibit magnetic behavior similar to ferrites, IONPs, and pure metal MNPs. Their superparamagnetic nature enables rapid response to AMFs and efficient heat generation, making them suitable for magnetic hyperthermia. Furthermore, their high saturation magnetization and magnetic anisotropy allow strong directional alignment under an applied field, an important feature for targeted drug delivery. Metallic alloy MNPs also offer advantages not typically found in other magnetic materials. They exhibit excellent chemical stability and strong oxidation resistance. Their magnetic anisotropy is temperature-dependent, as they can undergo a phase transition from a low-anisotropy cubic A1 phase (figure 2(I)) to a high-anisotropy (∼6,600 kJ m^−3^) L10 phase (figure 2(J)) with a face-centered tetragonal (FCT) crystal structure (figure 2(J)). In the A1 phase, Fe and Pt atoms are randomly distributed, whereas in the FCT phase, their positions along the vertical axis are well ordered. These properties have led to their use in high-density data storage media, such as hard disk drives. Other metallic alloy MNPs can adopt distinct crystal structures. For instance, FeCo exists in an ordered B2 phase at room temperature, consisting of two interpenetrating simple cubic sublattices with Fe atoms occupying one sublattice and Co atoms the other. Above 730 °C, it transitions to a disordered A2 phase in which Fe and Co atoms are randomly arranged [51].

Both physical and chemical synthesis methods are used to produce metallic alloy MNPs. Physical approaches, such as ball milling, sputtering, pyrolysis, radiolytic techniques, and sonochemical synthesis, generally break down bulk materials into nanoscale particles. Chemical approaches typically start from metal atoms or salts, using processes such as precipitation in basic solutions, microemulsion techniques (using water–oil–surfactant mixtures to form nanoparticles within microdroplets), hydrothermal or solvothermal synthesis in an autoclave, and thermal decomposition. Because the magnetic and structural properties of metallic alloy MNPs are sensitive to temperature, they are often stabilized during synthesis by incorporating secondary metallic elements such as Ag, Au, Sb, or Cu. These dopants can shift the ordering temperature at which phase transitions occur, reduce coercivity (often at the expense of magnetic stability), and influence particle morphology. For example, Ag tends to promote spherical shapes, whereas Au can produce columnar morphologies.

### 2.2. Magnetic properties of MNPs relevant to theranostics

#### 2.2.1. Size-dependent properties

Intrinsic magnetic properties of MNPs, such as anisotropy (K) and saturation magnetization (M_s_), are strongly size dependent. The Curie temperature, which reflects the strength of ferromagnetic exchange interactions, also varies with particle size due to changes in magnetic coupling between grains. As MNP size decreases, the high surface-area-to-volume ratio leads to a reduction in M_s_. This drop is primarily attributed to spin canting in the shell region: while spins in the core align with the applied magnetic field and retain the same magnetization as the bulk material, spins at the surface are often misaligned in random orientations, contributing zero net magnetization [52]. The balance between core and shell contributions—dictated by particle size and applied field strength—determines the effective magnetization. Several mechanisms have been proposed to explain surface spin canting effects [53]: (1) Symmetry breaking at the nanoparticle surface, leading to strong localized surface anisotropy; (2) Lattice distortion and variation of lattice constants in the crystal structure; (3) Surface disorder that reduces coordination number (broken bonds) and nearest-neighbor exchange interactions, altering the system’s energy minimum; (4) Presence of vacancies that disrupt parallel spin alignment; (5) Internal magnetic interactions that rotate spin orientation away from the applied field.

MNP size also influences the effective anisotropy (K_eff_), which comprises two main components: (1) Crystalline anisotropy (K_v_): the energy difference between the default magnetization direction and the direction aligned with the applied field; (2) Shape anisotropy (K_s_): inversely proportional to particle diameter and significant for asymmetric geometries (e.g., ellipsoids, rectangular prisms). K_s_ is negligible for symmetric shapes such as spheres, cubes, or regular octahedra because the contributions from different surfaces cancel out. Moreover, in materials with low spin–orbit coupling (e.g., transition metals and metal oxides) or where exchange anisotropy dominates over surface anisotropy, the shape anisotropy term becomes insignificant.

#### 2.2.2. Superparamagentism

The magnetic properties of nanoparticles are strongly influenced by particle size (volume). One key property is magnetic anisotropy, which governs the thermal stability of the magnetic moment. When particle dimensions shrink into the nanometer regime—as is the case for MNPs—the material can no longer support multiple magnetic domains. At a critical particle size, the entire particle becomes a single magnetic domain, and the corresponding particle diameter is known as the single-domain diameter (D_s_). D_s_ can be estimated using [42]:

\begin{eqnarray*}{D}_{s}=\frac{18\,{({A}_{{ex}}{K}_{u})}^{1/2}}{{\mu }_{0}{M}_{s}^{2}},\end{eqnarray*}

where A_ex_ is the exchange stiffness (in J/m), K_u_ is the crystalline anisotropy (in J/m^3^), and ${\mu }_{0}$ is the vacuum permeability.

In a single-domain particle, the magnetization naturally aligns along one of the crystal’s ‘easy’ axes to minimize total energy. As MNP size further decreases, the anisotropy energy (${K}_{u}V$) becomes comparable to or even smaller than the thermal energy (${k}_{B}T$). Below this threshold, thermal fluctuations can randomly flip the particle’s magnetic moment, similar to the behavior of spins in a paramagnetic material. This results in vanishing coercivity and defines the superparamagnetic state. This stage is classified as the superparamagnetic state for the MNP. The diameter of the MNP in this state (D_p_) can be estimated from the balance between thermal energy and crystalline anisotropy energy [42]:

\begin{eqnarray*}{D}_{p}={\left(\frac{48{k}_{B}T}{{K}_{u}V}\right)}^{1/3}\end{eqnarray*}

In biomedical applications, superparamagnetism offers distinct advantages: nanoparticles respond strongly to an applied magnetic field but exhibit no remanent magnetization once the field is removed. This ensures that their activity is confined to the period of magnetic field exposure, reducing unwanted aggregation or side effects. A prominent example is superparamagnetic iron oxide nanoparticles (SPIONs), typically composed of maghemite (*γ*-Fe_2_O_3_), magnetite (Fe_3_O_4_), or a mixture of both. When administered in controlled concentrations, SPIONs can perform a variety of biomedical functions, including [54](1) Enhancement of the hemoglobin formation process; (2) increasing lymphocyte production, thus improving the immune system; (3) improving conduction along nerve fibers; (4) creating essential enzymes and hormones in the body.

#### 2.2.3. Static magnetization models

The performance of MNPs in biomedical applications depends on their ability to respond to an externally applied magnetic field and to retain or lose their magnetization once the field is removed. An applied static magnetic field directly alters the magnetization of an MNP, and therefore, its magnetic moment, which determines the magnitude of the magnetic force it experiences. In addition, the 3D field distribution and spatial field gradients (dH/dx) strongly influence the trajectory and overall behavior of MNPs during magnetophoresis. These effects are particularly critical in magnetophoretic and drug/gene delivery applications, where precise control of particle motion is required.

Under a static DC field, magnetization behaviors of MNPs can be described by the Stoner–Wohlfarth model. This model assumes that each MNP is in a single magnetic domain. The total energy is the sum of the Zeeman energy (E_p_, from the applied field) and the anisotropy energy (E_k_, from crystalline anisotropy). In a zero field, the magnetization aligns along the anisotropy axis. When a field is applied, torque (**M** × **H**) causes the magnetization to rotate toward the field direction. The model assumes that the magnitude of the magnetization remains constant at all times. By minimizing the total energy, the equilibrium magnetization state of an MNP can be determined. Because the Stoner–Wohlfarth model only considers anisotropy and the external field, it ignores interparticle interactions. As a result, it is not suitable for concentrated MNP ensembles where dipole–dipole interactions dominate. These interactions depend on the interparticle distance and the relative magnetization orientations; strong coupling can restrict magnetization rotation, producing a ferromagnetic-like state with increased coercivity [42, 55, 56]. Furthermore, the model applies strictly to T = 0 K, neglecting thermal effects at higher temperatures.

For T > 0, thermal fluctuations must be included. The temperature dependence of an MNP’s magnetic moment can be described by the quantum mechanical Brillouin function, which depends on particle volume, the total angular momentum J (the sum of spin S and orbital angular momentum L), and the Landé g-factor. In the classical limit (S → ∞, J → ∞), the Brillouin function reduces to the Langevin function. The Langevin model describes the magnetization of a collection of non-interacting MNPs with no preferred anisotropy axis, assuming that the system remains in thermal equilibrium at all times under a DC or slowly varying AC magnetic field. For equilibrium to hold, the magnetization measurement time must be much longer than the nanoparticle’s relaxation time.

**Figure 3.**
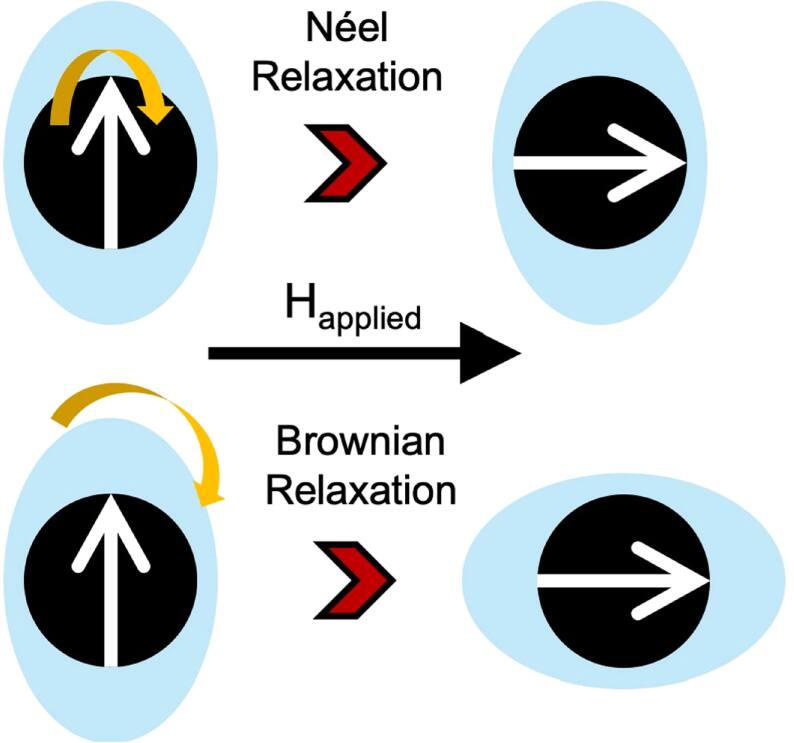
Néel and Brownian relaxations. ‘H_applied_’ represents the applied field. Original figure prepared by the authors.

Upon the removal of external magnetic field, magnetic moments of MNPs relax toward equilibrium states via two mechanisms (figure 3): (1) Brownian relaxation: both the magnetic moment and the particle’s anisotropy axis physically rotate within the medium; (2) Néel relaxation: only the magnetic moment rotates, while the anisotropy axis remains fixed. Both mechanisms have distinct relaxation times, and the dominant process depends on which time is shorter.

In the absence of an external magnetic field, the zero-field Néel and Brownian relaxation times are described by:

\begin{eqnarray*}{\tau }_{0}=\frac{{M}_{s}}{2\alpha \gamma {K}_{u}}{\mathrm{;}}\sigma =\frac{{K}_{u}V}{{k}_{B}T}{\mathrm{;}}{\tau }_{N,0}=\frac{\sqrt{{\mathrm{\pi }}}}{2}{\tau }_{0}\frac{\exp \,(\sigma )}{{\sigma }^{1/2}}\end{eqnarray*}

\begin{eqnarray*}{\tau }_{B,0}=\frac{{3V}_{h}\eta }{{k}_{B}T},\end{eqnarray*}

where ${\tau }_{0}$ is the atomic attempt time, which is a characteristic of the material. M_s_ is the saturation magnetization, $\alpha $ is the Gilbert damping constant, and $\gamma $ is the gyromagnetic ratio. K_u_ is the anisotropy constant, ${V}_{h}$ is the hydrodynamic volume, and $\eta $ is the viscosity of the liquid surrounding the MNP. Equation (3) describes the Néel relaxation time, and equation (4) describes the Brownian relaxation time. The effective zero-field relaxation time ${\tau }_{{eff},0}$ is governed by both Brownian and Néel relaxation processes. Assuming the Néel and Brownian relaxations are independent, a commonly used model to calculate the ${\tau }_{{eff},0}$ is given as:

\begin{eqnarray*}\frac{1}{{\tau }_{{eff},0}}=\frac{1}{{\tau }_{B,0}}+\frac{1}{{\tau }_{N,0}}\end{eqnarray*}

However, equation (5) is limited by its assumption of independence between the two relaxation mechanisms and equilibrium states at every time step. This restricts its applicability under DC fields or slowly varying AC fields, where the MNP reaches an equilibrium state at all times. This model cannot describe non-equilibrium conditions where relaxation is absent.

#### 2.2.4. Dynamic magnetization models

Dynamic magnetization models are well-suited for capturing MNPs’ magnetization behaviors under time-varying fields. This is especially relevant for the applications of MNPs in magnetic hyperthermia, MRI, and MPI, where fast-changing AMFs and/or non-zero gradient fields are applied. Under these conditions, the excitation field simultaneously drives Néel and Brownian relaxation processes, which together determine the dynamic magnetization of the particles. Consequently, the AC magnetic hysteresis loops of MNPs are broader than their DC counterparts, reflecting increased energy dissipation per cycle and resulting in enhanced heat generation (i.e., higher SAR) for magnetic hyperthermia. In MPI, the nonlinear dynamic magnetization produces higher-order harmonics of the drive field, which are fundamental to signal generation and image reconstruction. In MRI, radio-frequency excitation perturbs the dipolar fields generated by nearby MNPs, influencing proton spin precession and altering T1/T2 relaxation pathways used for contrast formation.

When the AMF amplitude is low, MNP’s magnetization changes linearly with the applied field. The linear response is described by a DC susceptibility (${\chi }_{0}$) in the Debye model [50, 57]:

\begin{eqnarray*}{\chi }_{0}=\frac{M\,(H)}{H}=\frac{{M}_{s}}{H}{\mathscr{L}}{\mathscr{(}}H),\end{eqnarray*}

where ${\mathscr{L}}{\mathscr{(}}H)$ is the Langevin function and ${\chi }_{0}$ is the magnetic susceptibility. The Debye model is commonly used to describe the magnetic relaxation of MNPs under low-amplitude AMFs, where the M–H relationship remains linear. Yoshida *et al* [58] extended this model to account for Brownian relaxation in the presence of a large DC field, deriving a field-dependent Brownian relaxation time from the Langevin function and the Fokker–Planck equation. Dieckhoff *et al* [59] proposed a field-dependent Néel relaxation model by fitting experimental data. Both studies showed that relaxation times decrease with increasing field strength, with Néel relaxation exhibiting a stronger field dependence.

Another model developed on similar lines to clarify the MNP’s magnetization behavior in the presence of varying fields is based on the Landau–Lifshitz–Gilbert (LLG) equation. This differential equation describes the precessional motion of magnetizations in a crystalline lattice under the influence of damping and an external field. The LLG equation does not consider the medium around the MNP, which means the Brownian relaxation is ignored. The LLG equation is used in software like OOMMF and MUMAX, and primarily as a tool to capture magnetization dynamics of materials in magnetic recording [60–64].

Given the need to model coupled dynamics with both Néel and Brownian relaxations, models have been established to capture dynamics for the coupled relaxations under different fields. A stochastic Langevin equation-based model is used to predict the dynamic magnetizations of MNPs when combined with relaxation processes. Although this model is an improvement in accounting for the coupling of relaxation mechanisms, it is computationally intensive, and the simulation of magnetization for a large ensemble of particles is challenging. In this model, the easy axis (anisotropy) and magnetization are two separate unit vectors in two different directions. In 2018, Weizenecker reported the Fokker–Planck equations for the coupled relaxations, based on the coupled Langevin equations [65]:

\begin{eqnarray*}\widehat{{\boldsymbol{n}}}={N}_{x}\widehat{{\boldsymbol{x}}}+{N}_{y}\widehat{{\boldsymbol{y}}}{\boldsymbol{+}}{N}_{z}\widehat{{\boldsymbol{z}}}{\boldsymbol{=}}{N}_{\rho }{\widehat{{\boldsymbol{e}}}}_{{\boldsymbol{\rho }}}+{N}_{\theta }{\widehat{{\boldsymbol{e}}}}_{{\boldsymbol{\theta }}}{\boldsymbol{+}}{N}_{\phi }{\widehat{{\boldsymbol{e}}}}_{{\boldsymbol{\phi }}}\end{eqnarray*}

\begin{eqnarray*}\widehat{{\boldsymbol{m}}}={U}_{x}\widehat{{\boldsymbol{x}}}+{U}_{y}\widehat{{\boldsymbol{y}}}{\boldsymbol{+}}{U}_{z}\widehat{{\boldsymbol{z}}}{\boldsymbol{=}}{U}_{r}{\widehat{{\boldsymbol{e}}}}_{{\boldsymbol{r}}}+{U}_{\vartheta }{\widehat{{\boldsymbol{e}}}}_{{\boldsymbol{\vartheta }}}{\boldsymbol{+}}{U}_{\varphi }{\widehat{{\boldsymbol{e}}}}_{{\boldsymbol{\varphi }}},\end{eqnarray*}

where the uniaxial anisotropy (${K}_{u}\widehat{{\boldsymbol{n}}}$) and the magnetization (${M}_{S}\widehat{{\boldsymbol{m}}}$)are defined in a spherical coordinate system. The unit vectors in the direction of magnetization and easy axis are denoted as $\widehat{{\boldsymbol{m}}}$ and $\widehat{{\boldsymbol{n}}}$, respectively. N and U represent the magnitudes in different directions for the anisotropy and moment, respectively. Based on the coupled Langevin equations, the coupled Fokker-Planck equation can be expressed as:

\begin{eqnarray*}\begin{array}{ccc}\frac{\partial P}{\partial t} &amp; = &amp; -\frac{1}{2{\tau }_{B0}}\frac{1}{\sin \vartheta }\frac{\partial }{\partial \vartheta }\left[\frac{2{K}_{u}{V}_{C}}{{k}_{B}T}{U}_{r}{U}_{\vartheta }\sin \vartheta P-\sin \vartheta \frac{\partial F}{\partial \vartheta }\right]\\ &amp; &amp; -\frac{1}{2{\tau }_{B0}}\frac{1}{\sin \vartheta }\frac{\partial }{\partial \varphi }\left[\frac{2{K}_{u}{V}_{C}}{{k}_{B}T}{U}_{r}{U}_{\varphi }P\right]\\ &amp; &amp; -\frac{1}{2{\tau }_{N0}}\frac{1}{\sin \theta }\frac{\partial }{\partial \theta }\left[\frac{{\mu }_{0}{M}_{S}{V}_{C}}{\alpha {k}_{B}T}\left(\alpha {H}_{\theta }-{H}_{\phi }\right)\sin \theta P\right.\\ &amp; &amp; \left.+\frac{2{K}_{u}{V}_{C}}{\alpha {k}_{B}T}{N}_{\rho }\left(\alpha {N}_{\theta }-{N}_{\phi }\right)\sin \theta P-\sin \theta \frac{\partial P}{\partial \theta }\right]\\ &amp; &amp; -\frac{1}{2{\tau }_{N0}}\frac{1}{\sin \theta }\frac{\partial }{\partial \phi }\left[\frac{{\mu }_{0}{M}_{S}{V}_{C}}{\alpha {k}_{B}T}\left(\alpha {H}_{\phi }+{H}_{\theta }\right)P\right.\end{array}\end{eqnarray*}

\begin{eqnarray*}\begin{array}{ccc} &amp; &amp; \left.+\frac{2{K}_{u}{V}_{C}}{\alpha {k}_{B}T}{N}_{\rho }\left(\alpha {N}_{\phi }+{N}_{\theta }\right)P\right]\\ &amp; &amp; +\frac{1}{2{\tau }_{B0}}\frac{1}{{\sin }^{2}\vartheta }\frac{{\partial }^{2}P}{\partial {\varphi }^{2}}+\frac{1}{2{\tau }_{N0}}\frac{1}{{\sin }^{2}\theta }\frac{{\partial }^{2}P}{\partial {\phi }^{2}}\end{array}\end{eqnarray*}

where P is the probability density function (PDF) for a collection of MNPs. It represents the distribution of locations on a unit sphere where the magnetization is deemed to point. The coupled Fokker-Planck equation computes the angular distribution of the particles, considering the contributions of the anisotropy axis and magnetization in the model.

### 2.3. Surface modification and functionalization of MNPs for biomedical applications

MNPs have become a valuable tool for a wide range of biomedical applications and therapies owing to their exceptional magnetic properties, small size, and low immunogenicity. Their ability to be precisely controlled by an external magnetic field, along with their nanoscale size, makes them highly effective for targeted drug delivery, MRI, and hyperthermia treatment [66, 67]. However, the successful implementation of MNPs in biological systems faces several challenges. Unmodified MNPs tend to aggregate due to magnetic dipole–dipole interactions and remanent magnetization, combined with insufficient surface charge (low zeta potential) to provide adequate repulsive forces. Moreover, unmodified MNPs often exhibit poor colloidal stability in physiological conditions and rapid clearance from the bloodstream by the reticuloendothelial system [68]. To address these limitations, surface modification and functionalization methods have been adopted. These strategies not only contribute to stabilizing the nanoparticles but also enable the conjugation of therapeutic agents, targeting ligands, and diagnostic markers that increase the multipurpose use of nanoparticles in the biomedical field. The surface modification of MNPs can significantly enhance their biocompatibility, circulation time, and targeting efficiency while reducing their toxicity and immunogenicity [69]. Coatings applied to MNPs can be categorized into three main types: organic coatings, inorganic coatings, and cell membrane coatings, each offering unique properties for specific biomedical applications [70].

#### 2.3.1. Organic coatings

##### 2.3.1.1. Natural biopolymer coatings

**Figure 4.**
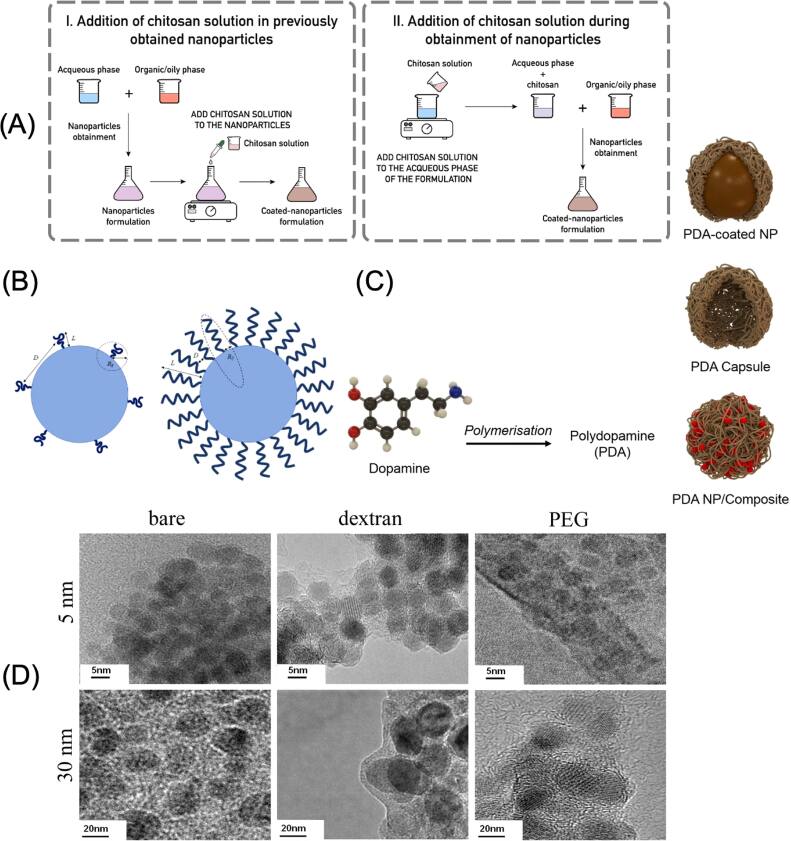
Representative strategies for surface coating of MNPs. (A) The two typical methods for preparing chitosan-coated nanoparticles, either by adding chitosan after nanoparticle synthesis or by incorporating it during formation. (B) Schematic of PEG confirmations on MNP surfaces. (C) Formation of PDA coatings from dopamine polymerization and resulting PDA-based nanostructures. (D) TEM images comparing bare, dextran-coated, and PEG-coated MNPs at different magnifications. (A) reprinted with permission from [79], copyright 2019 Elsevier B.V. (B) reprinted from [80], licensed under CC BY 4.0. (C) reprinted from [81], licensed under CC BY 4.0. (D) reprinted from [82], licensed under CC BY 4.0.

Natural biopolymer coatings represent a versatile class of surface modifications that enhance MNP functionality. These biopolymers, which are derived from natural sources, are biocompatible, biodegradable, non-toxic, and can be easily modified with active substances–therapeutic or diagnostic [71]. The selection of natural biopolymers for MNP coating is primarily guided by their unique chemical structures and biological properties, which enable specific interactions with both the nanoparticle surface and the biological environment. An example of this type of biopolymer is chitosan, a cationic polysaccharide structurally like cellulose, but with the hydroxyl (-OH) group at the C_2_ position replaced by an acetamido (-NHCOCH_3_) group. It consists of glycosidic linkages of 2-amino-2-deoxy-D-glucose and is derived from chitin, a natural component of crustacean shells. This polymer offers multiple functionalities, including antimicrobial activity and the ability to act as a bearer of other chemical entities through its primary amine groups. A notable characteristic of chitosan is its pH-dependent solubility; while insoluble at neutral or basic pH, it becomes water-soluble in acidic conditions due to the protonation of its amino groups. The coating process of chitosan onto MNPs can be achieved through two distinct approaches, as illustrated in figure 4(A): post-synthesis modification, where chitosan solution is added to pre-formed nanoparticles, or *in situ* coating during nanoparticle formation. The coating mechanism relies on electrostatic interactions, where the positively charged ammonium groups of chitosan interact with negative charges on the nanoparticle surface under acidic conditions. Beyond its basic properties, chitosan is biodegradable, breaking down into nontoxic amino sugar degradation products. It is also cost-effective, exhibits low immunogenicity, and rarely induces allergic reactions [72]. These properties, combined with its ability to adsorb harmful substances and its antibacterial and immunostimulatory qualities, make it particularly valuable for magnetic separation and affinity protein purification applications [73–76].

Dextran, a hydrophilic polymer composed of glucose monomers linked via α-1,6-glycosidic bonds, is widely valued for its ability to enhance nanoparticle stability in aqueous environments [77]. In alkaline solutions, dextran adsorbs onto MNPs through noncovalent interactions; however, this relatively weak binding remains a key limitation of dextran coatings. The stability of dextran-coated MNPs in biological tissues is particularly notable, as human cells lack dextranase, the enzyme responsible for dextran degradation [72]. Recent developments have focused on carboxymethyl dextran (CMD), a dextran derivative that introduces both hydroxyl and carboxyl functional groups. These groups enable easier chemical modification while preserving the polymer’s excellent biocompatibility, biodegradability, and high water solubility [78].

Polyethylene glycol (PEG) modification represents a crucial advancement in MNP surface engineering. This synthetic linear polymer exhibits hydrophilic properties and can be functionalized with terminal groups such as amine (-NH_2_), thiol (-SH), and carboxyl (-COOH), allowing chemical conjugation to nanoparticles and biomolecules. Like PEGylated proteins, PEG coatings on nanoparticles help prevent aggregation, opsonization, and phagocytosis. When grafted onto the surface of MNPs, PEG forms a hydrophilic shield that minimizes immune recognition, thereby significantly prolonging their circulation time in the bloodstream [83]. The effectiveness of PEG coating depends on its surface arrangement, as shown in figure 4(B), where PEG chains can adopt either a mushroom configuration when sparsely distributed on the surface or a brush configuration when densely packed. These distinct conformations directly influence the stealth properties and biological interactions of the PEG-coated MNPs. The distinctive properties of PEG, including water solubility, biocompatibility, flexibility, nontoxicity, and nonantigenic behavior, make it particularly valuable for biomedical applications. Furthermore, PEG-coated MNPs can demonstrate enhanced targeting of specific tissues or cells, but their renal elimination is dependent on maintaining a hydrodynamic diameter below 5.5 nm, which is crucial for kidney filtration and influences their theragnostic potential [84]. These properties have made PEG-coated MNPs highly effective for detecting and treating various diseases, including cancer, cardiovascular disease, and neurological disorders [72, 85]. The ‘stealth’ properties conferred by PEG coating have been shown to reduce nanoparticle recognition by macrophages and enhance their accumulation in target tissues through the enhanced permeability and retention (EPR) effect [86].

More recent developments in biopolymer coatings include polydopamine (PDA), which has emerged as a versatile coating material inspired by mussel adhesive proteins. PDA-coated MNPs demonstrate remarkable versatility, particularly in cancer therapy applications, where they enhance photothermal properties while providing a robust platform for further surface modifications [87]. As illustrated in figure 4(C), the versatility of PDA coating is evident in its various structural forms: PDA-coated nanoparticles, PDA capsules, and PDA nanoparticle composites, each providing unique advantages for different applications. It is reported that under near-infrared (NIR) laser irradiation (808 nm, 4.6 W cm^−2^), PDA-coated MNPs generate localized hyperthermia with temperature increases up to 43 °C within 2 min [88]. This efficient photothermal response enables tumor ablation at relatively low laser power densities while minimally affecting surrounding healthy tissues. Recent studies have shown that PDA-coated MNPs are especially effective for photothermal therapy and tumor resection, exhibiting minimal side effects and improved therapeutic outcomes [89–91]. The effectiveness of these coating strategies is verified through transmission electron microscopy (TEM), as shown in figure 4(D), where both dextran and PEG coatings form uniform layers on 5 nm and 30 nm MNPs, with coating thicknesses of approximately 2 nm and 5 nm, respectively, demonstrating successful surface functionalization at different nanoparticle size scales. While biopolymer coatings offer excellent biocompatibility, other coating materials can provide additional physicochemical properties beneficial for specific applications.

##### 2.3.1.2. Synthetic polymer coatings

Beyond natural biopolymers, synthetic polymers represent another significant category of available surface modifications for MNPs, offering precisely controlled properties and enhanced functionality for biomedical applications. The coating process for synthetic polymers can be achieved through several approaches, including *in situ* polymerization during nanoparticle synthesis, post-synthesis grafting, and layer-by-layer assembly. Each method offers distinct advantages in terms of coating uniformity, thickness control, and stability. The incorporation of synthetic polymers onto MNP surfaces can be achieved through two primary approaches: the ‘grafting from’ method, where polymerization occurs directly from the MNP surface, and the ‘grafting to’ method, where pre-formed polymers are attached to the surface [92]. These can be engineered with specific molecular weights, architectures, and functional groups to optimize their performance in biological systems while addressing key challenges, such as particle agglomeration, core oxidation, and potential toxicity [93].

Temperature-responsive synthetic polymers have emerged as particularly valuable for use in controlled drug delivery applications [94–96]. Among these, Poly(N-isopropylacrylamide) (PNIPAAm) exhibits a low critical solution temperature (LCST) of approximately 32 °C, undergoing a reversible phase transition that enables thermally-triggered drug release and cellular uptake mechanisms. This temperature-dependent behavior makes PNIPAAm-coated MNPs particularly effective for controlled drug delivery applications where hyperthermia can be employed as a trigger mechanism [97].

Several synthetic polymers have been developed specifically to enhance MNP stability and biocompatibility. Polyvinylpyrrolidone (PVP) has gained significant attention for its exceptional stabilizing properties. When applied to MNPs, PVP forms a protective layer that enhances colloidal stability and reduces protein adsorption, crucial features for maintaining nanoparticle dispersion in physiological environments. The polymer’s amphiphilic nature enables it to act as both a stabilizer and a bridge for further functionalization, making PVP-coated MNPs versatile platforms for various biomedical applications [98].

Biodegradable synthetic polymers offer unique advantages through their controlled degradation profiles. Poly(lactic-co-glycolic acid) (PLGA), an FDA-approved polymer, enables sustained drug release over periods ranging from weeks to months, with degradation rates that can be precisely tuned by adjusting the ratio of lactic to glycolic acid monomers. This property, combined with the polymer’s excellent biocompatibility, has led to significant advances in targeted drug delivery systems and tissue engineering applications [99].

Stimuli-responsive synthetic polymers provide additional control over MNP behavior in biological environments. Polyacrylic acid (PAA) introduces pH-responsive behavior through its carboxylic acid groups, enabling targeted drug release in specific physiological environments. This is particularly valuable for cancer therapy applications, as tumor microenvironments possess pH levels that typically differ from healthy tissue [100, 101]. Similarly, polypropylene sulfide (PPS) exhibits unique reactive oxygen species (ROS)-responsive properties, making it a promising material for targeted drug delivery and cancer therapeutics. When used as a coating for MNPs, PPS may enable ROS-triggered drug release, enhancing site-specific delivery in oxidative tumor microenvironments [102].

For tissue engineering applications, structural synthetic polymers such as poly-3-hydroxybutyrate (PHB) have shown promise when combined with magnetite. PHB enables the creation of electrospun composite scaffolds that offer unique advantages due to their biocompatibility and structural properties [103]. The combination of PHB with MNPs has enabled the development of magnetically responsive scaffolds that can enhance cell adhesion and tissue regeneration processes [104]. The selection of a coating strategy depends largely on the specific polymer properties and requirements for the intended application. Recent advances in synthetic polymer coating techniques have enabled the development of multi-functional nanoplatforms that combine magnetic targeting with controlled release mechanisms, imaging capabilities, and tissue-specific targeting [92, 102, 103].

#### 2.3.2. Inorganic coatings

Inorganic coatings offer an alternative strategy for MNP surface modification, primarily using materials such as silica, gold, and carbon [105]. Silica coatings, in particular, have attracted significant attention due to their exceptional chemical stability, versatile surface chemistry, and ability to protect the magnetic core [106]. Core–shell MNP/silica structures produce water-soluble, colloidally stable, and photostable particles. This stability arises from two main mechanisms: strong interactions between cations and the silicate layer at silica–water interfaces under basic conditions, and relatively weak van der Waals interactions owing to silica’s lower Hamaker constant [107, 108]. The silica shell not only prevents core degradation but also presents abundant surface silanol groups that readily react with alcohols and silane coupling agents, forming stable dispersions in nonaqueous solvents. These silanol groups serve as robust anchors for covalent ligand attachment, with strong binding affinities that minimize desorption [109]. Furthermore, functionalization with carboxyl, thiol, or amine groups further broadens their biomedical applications, including bioseparation, enzyme immobilization, and diagnostic testing.

Gold coatings provide another route to enhance MNP functionality, offering unique optical and surface properties that enable theranostic applications. The gold shell protects the magnetic core while improving conductivity, optical properties, biocompatibility, and chemical stability. These properties can be precisely tuned by adjusting particle size, shell thickness, shape, charge, and surface modifications [6]. A notable feature of gold-coated MNPs is their surface plasmon resonance, which facilitates strong absorption and scattering of NIR light. This effect enables surface-enhanced Raman scattering (SERS) and deeper tissue penetration, making these particles especially suitable for biomedical imaging and therapy. Gold-coated MNPs are versatile for applications such as bioseparation, electrochemical and optical sensing, and targeted drug delivery. Their potential in photothermal tumor therapy is particularly promising, as NIR-absorbing gold-modified MNPs exhibit reduced cytotoxicity toward healthy cells [72]. Additionally, gold’s high x-ray attenuation coefficient enhances computed tomography (CT) contrast, enabling dual-mode MRI–CT imaging [110].

While gold coatings excel in photothermal therapy and molecular conjugation, the search for materials offering both high thermal efficiency and extensive surface functionality has led to significant advances in carbon-based coatings. Graphene and carbon nanotubes (CNTs) have introduced new possibilities for MNP applications due to their extraordinary physicochemical properties. Graphene coatings provide exceptional mechanical strength and chemical stability while offering a large surface area (theoretical value of 2,630 m^2^ g^−1^) for drug loading through π-π stacking interactions. The high thermal conductivity of these carbon materials enables efficient heat transfer during magnetic hyperthermia treatment, significantly enhancing therapeutic outcomes in cancer treatment. CNT coatings, in particular, demonstrate remarkable electrical conductivity and mechanical flexibility, making them ideal for biosensing applications and controlled drug release triggered by external stimuli [111, 112]. The sp^2^ hybridized carbon structure also allows for multiple functionalization strategies, enabling the attachment of various therapeutic and targeting molecules while maintaining the inherent properties of both the carbon coating and the magnetic core. Despite the advantages of both biopolymer and inorganic coatings, researchers have sought more biomimetic approaches to enhance the biological interactions of MNPs, leading to the development of novel coating strategies.

#### 2.3.3. Cell membrane coatings

**Figure 5.**
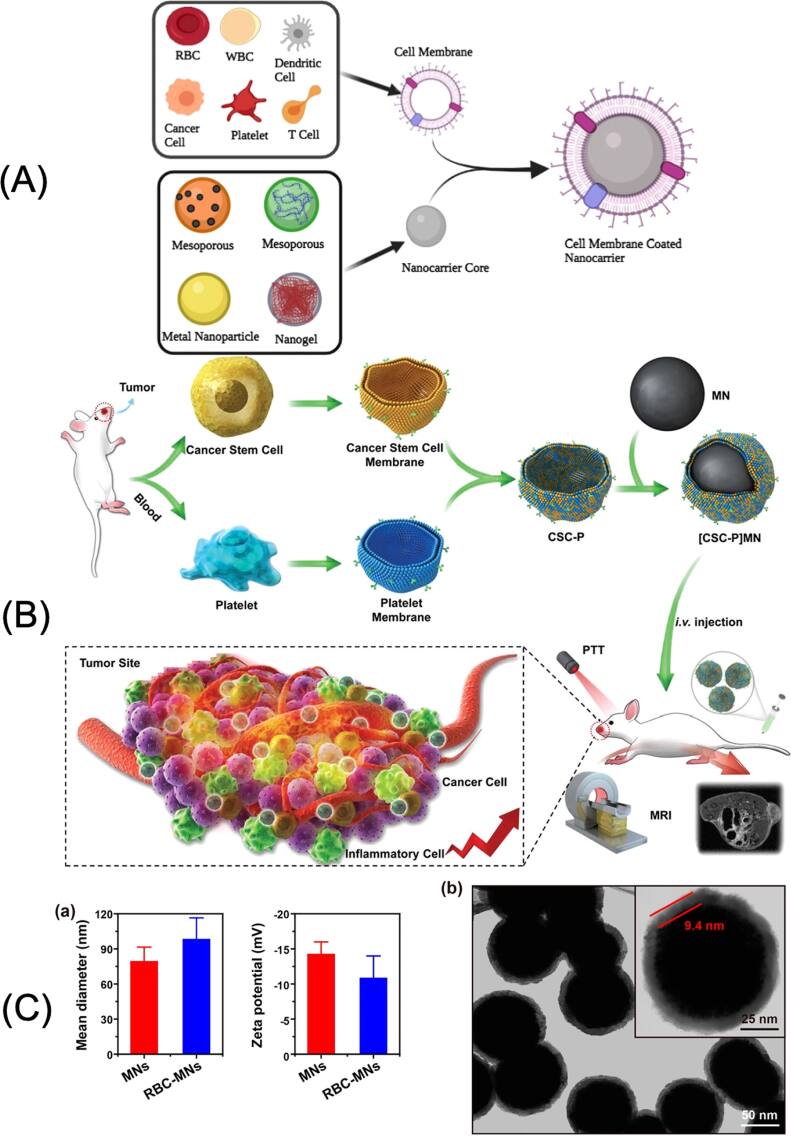
(A) Schematic illustration of various cell membrane sources and nanocarrier cores used in cell membrane coating technology; (B) Fabrication of platelet-cancer stem cell hybrid membrane-coated MNPs and their application for tumor imaging and photothermal therapy; (C) Characterization of red blood cell (RBC) membrane-coated MNPs. (a) showing size distribution, zeta potential, and (b) TEM imaging (scale bar = 50 nm). (A) reprinted from [117], licensed under CC BY 4.0. (B) reprinted with permission from [115], copyright 2019 WILEY-VCH Verlag GmbH & Co. KGaA, Weinheim. (C) reprinted with permission from [114], copyright 2017 American Chemical Society.

As shown in figure 5(A), the cell membrane coating technology for MNPs involves several steps in isolating the biological cell membrane and then attaching it to the MNP core to form a shell structure. These biomimetic structures combine the magnetic responsiveness of the MNP core with the biological functionality of the cell membrane shell, enabling the particles to navigate effectively within biological environments. For instance, red blood cell membrane-coated MNPs are showcased for their unique property of hiding from the immune system [113]. As demonstrated in figure 5(C), red blood cell membrane-coated MNPs are characterized by distinct size distributions and zeta potentials compared to uncoated MNPs, with TEM imaging revealing a uniform membrane coating [114]. These nanoparticles are biocompatible and remain functional for extended periods due to the ability to design them in a manner that resembles native red blood cells, thus avoiding activation of immune responses that would lead to the elimination of the nanoparticles from circulation. Among the various cell membrane sources, platelet membrane coatings have emerged as a particularly promising approach due to their unique targeting capabilities. As illustrated in figure 5(B), Bu *et al* advanced this concept further by developing a hybrid platelet-cancer stem cell membrane-coated iron oxide MNP ([CSC-P]MNP) for enhanced photothermal therapy of head and neck squamous cell carcinoma (HNSCC) [115]. Compared to single-cell membrane-coated nanoparticles, these hybrid [CSC-P]MNPs demonstrated superior characteristics, including prolonged circulation times, enhanced targeting abilities, and excellent photothermal properties. More recently, Tavakoli *et al* explored the potential of platelet membrane-coated SPIONs for cancer therapy, combining targeted paclitaxel delivery with magnetic hyperthermia [116]. This dual-therapeutic approach demonstrated the potential to minimize chemotherapy’s side effects while maximizing therapeutic outcomes.

In pursuit of enhanced tumor targeting, stem cells, particularly mesenchymal stem cells (MSCs), represent another promising source for cell membrane coatings due to their unique properties and versatile applications. MSCs offer significant advantages, including ease of acquisition, isolation from various tissue types, and inherently low immunogenicity [113, 118]. This natural tumor tropism makes MSC membrane-coated nanoparticles highly effective for anti-tumor applications. In a pioneering study, Kim *et al* developed MSC membrane-coated iron oxide nanoparticles (MNVs) that showed remarkable potential for treating ischemic stroke [119]. Specifically, these MNVs played crucial roles in promoting angiogenesis, anti-inflammatory responses, and anti-apoptosis, leading to significant reductions in infarction volume.

Complementing these approaches, white blood cells offer unique advantages as membrane sources due to their integral role in the immune system and their ability to actively transition between intravascular and extravascular tissues. White blood cell membrane-coated nanoparticles (WCNPs) leverage the cells’ native membrane proteins to facilitate active migration, enabling distinctive targeting capabilities for treating diseases such as cancer and inflammation [120, 121]. In a notable advancement, Liu *et al* developed an innovative biomimetic polymer magnetic nanocarrier (PLGA-ION-R837@M) coated with lipopolysaccharide-treated macrophage membranes for targeting tumor-associated macrophages (TAMs) [122]. They successfully demonstrated significant potential in enhancing anticancer immunotherapy through the targeted modulation of TAMs in the tumor microenvironment.

Recent advances in this area include the work by Li *et al*, who developed cancer cell membrane-coated MNPs for dual-modal imaging and photodynamic therapy [123]. The cancer cell membrane coating significantly improved both biocompatibility and cellular uptake, demonstrating enhanced anti-tumor effects both *in vitro* and *in vivo*. This capability, combined with magnetic responsiveness, is considered a breakthrough in theranostics since the coatings mimic the biological targeting ability of the cell membrane while enabling magnetic guidance. These coating technologies have opened new possibilities in targeted drug delivery, where the encapsulated agents are released selectively at the site of interest and only at the site of interest, such as tumors, thereby causing minimal damage to surrounding healthy tissues [6]. In addition to drug delivery, the biomimetic properties of these coatings would enable the integration of the system into living biological systems, opening the gates for complex imaging and elegant therapeutic methods. For example, since these coatings are flexible, MNPs may be used for multiple functions, such as drug delivery, imaging, and therapy, all at once. The versatility of replicating natural biological behaviors and functions makes cell membrane coatings important for the next generation of biomedical applications.

#### 2.3.4. Limitations of coating methods

Table 1 summarizes the different types of coatings that can be applied onto MNPs as reviewed in this work. While both biopolymer and inorganic coatings have demonstrated significant success in various biomedical applications, their synthetic nature still poses limitations in fully mimicking complex biological interactions. Biopolymer coatings, despite their biocompatibility, may not completely replicate the sophisticated cell–cell recognition mechanisms. Meanwhile, inorganic coatings, though stable and multifunctional, can still face challenges in evading immune system recognition. These limitations have driven researchers to explore more biomimetic approaches that could better simulate natural cellular interactions. This pursuit has led to the development of cell membrane coating technology, representing a groundbreaking strategy in MNP surface modification. Cell membrane coating represents an innovative approach to MNP surface modification. Natural cell membranes are extracted from biological cells such as red blood cells, cancer cells, or immune cells through a carefully controlled hypotonic treatment and purification process [124]. The extraction methodology preserves crucial membrane proteins and surface markers that are essential for cellular recognition and immune evasion [113]. These biomimetic coatings enable interaction with target cells. The source of the cell membrane significantly influences the functional properties of the coated MNPs. Red blood cell membranes have gained particular attention due to their naturally long circulation properties and immune-evading capabilities. In 2011, Zhang *et al* showed that red blood cell membrane-coated nanoparticles exhibited a circulation half-life of 39.6 h, compared to 15.8 h for polyethylene glycol-coated particles [125]. Cancer cell membrane coatings provide unique advantages through their homotypic targeting ability, where the retained surface antigens enable preferential accumulation in tumor tissues through cancer cell-specific recognition [120]. This homotypic targeting has demonstrated up to 40% higher tumor accumulation compared to non-cancer cell membrane-coated counterparts. Additionally, macrophage membrane coatings have shown promise in targeting inflammatory sites, leveraging their natural migration properties to enhance therapeutic delivery [126].

**Table 1.** Different types of coatings for MNPs.

Main category	Sub-category	Material	Applications	References
Organic Coatings	Natural Biopolymers	Chitosan	Drug delivery, Protein therapeutics	[127, 128]
		Dextran	Drug delivery, diagnostic imaging	[129]
		PDA	Cancer therapy, tumor ablation	[88, 90]
		PEG	Drug delivery, disease diagnosis, cancer treatment	[72, 85, 130]
	Synthetic Polymers	PVP	Drug delivery, disease diagnosis, cancer treatment	[98, 131, 132]
		PNIPAAm	Cancer therapy, targeted drug delivery, tumor treatment	[133–135]
		PAA		[136, 137]
		PPS		[102]
		PLGA	Drug delivery, tissue regeneration, disease treatment	[99, 138]
		PHB		[103, 104]
Inorganic Coatings	Metal-based	Silica	Disease diagnostics, therapeutic delivery	[139]
		Gold	Cancer therapy, medical imaging, disease diagnosis	[140, 141]
	Carbon-based	Graphene	Cancer treatment, medical imaging, therapeutic delivery	[142]
Cell Membrane Coatings	Blood Cell-derived	Red Blood Cell membrane	Disease treatment, inflammation therapy, immune therapy	[114, 143, 144]
		White Blood Cell membrane		[122, 145, 146]
	Tissue Cell-derived	Cancer cell membrane	Cancer therapy, tumor treatment	[115, 123, 147]
		MSC membrane		[119]
	Platelet derived	Platelet membrane	Cardiovascular treatment, cancer therapy	[115, 116, 148]

The versatility of cell membrane coatings extends beyond simple immune evasion. Recent studies have demonstrated their effectiveness in multimodal therapy applications. The incorporation of platelet membrane coatings has shown remarkable targeting efficiency for cardiovascular applications, with studies by Li *et al* demonstrating specific binding to damaged vessel walls and atherosclerotic plaques [149]. These coatings retain the complex protein structures and signaling molecules present on native platelets, enabling natural targeting mechanisms that synthetic coatings cannot replicate. Furthermore, cell membrane coatings provide unique opportunities for personalized medicine approaches. By utilizing patient-derived cell membranes, researchers have developed customized therapeutic platforms that minimize immune responses while maximizing targeting efficiency [150, 151]. The combination of magnetic targeting capabilities with the intrinsic biological properties of cell membranes has enabled precise spatial control over drug delivery while maintaining extended circulation times and reduced immunogenicity. Recent advances in membrane fusion techniques have also allowed for the development of hybrid membrane coatings, combining the advantages of multiple cell types to create more sophisticated therapeutic platforms [152, 153].

Although these biomimetic coatings markedly improve immune evasion and circulation time, they still provide limited control over precise tumor selectivity, as they rely primarily on natural homotypic or cell-type-specific interactions [154]. To further enhance targeting precision, active targeting has emerged as a complementary strategy in which MNPs are functionalized with ligands, such as folic acid, peptides, antibodies, and nucleic acid aptamers, which are enabled to recognize receptors that are highly expressed on cancer cells [155, 156]. These ligand–receptor interactions facilitate selective attachment and receptor-mediated internalization, enabling significantly greater tumor-specific uptake than passive or coating-mediated accumulation alone [157]. Active targeting can also help reduce off-target interactions within healthy tissues, complementing the circulation and stealth advantages provided by existing coating methods. Although optimization is still needed to ensure ligand stability and efficacy *in vivo*, this approach offers an additional level of specificity that can further refine the therapeutic and diagnostic performance of MNP-based platforms [158, 159].

## 3. Applications in cancer therapy

### 3.1. MNPs for hyperthermia therapy

The use of MNPs in hyperthermia has been the focus of multidisciplinary research in recent years [160–163]. Cancer cells are found to exhibit a unique trait of lower heat tolerance than healthy cells [164, 165]. Temperatures of 40 °C–44 °C can cause irreversible internal damage to cancer cells that affects their digestive functions. This vulnerability provides the ability to treat a variety of cancers using the hyperthermia method. MNPs can heat up to these temperatures by utilizing external AC magnetic fields, which provide advantages over other thermal treatments, such as microwaves [166], as this technology does not negatively affect adjacent healthy cells. Thus, the use of locally introduced targeted MNPs that affect primarily tumorous cells is of great interest.

The fundamental mechanisms of heat generation by MNPs are hysteresis losses, as well as Brownian and Néel relaxations [167]. Hysteresis losses are related to the hysteresis curve of ferromagnetic materials. The area within this curve is proportional to the amount of energy the MNP loses, referred to as hysteresis losses. Brownian relaxation is the physical rotation of the whole particle, while Néel relaxation is the internal rotation of the magnetic moment, as discussed above [165]. The domination of each relaxation mechanism depends on the applied field frequency and the size of the particle. The rotation of these particles causes an expenditure of energy, which comes in the form of heat [168, 169]. Long rotation times and large particle sizes give off more heat than short times and small particles. The heat generated by these MNPs is referred to as the specific absorption rate (SAR) [170].

MNPs have been exploited for hyperthermia applications for their superparamagnetic behavior, biocompatibility, and thermal abilities [167]. Superparamagnetism is ideal for hyperthermia applications as it allows for complete magnetization when an external field is applied and maximal dissipation of magnetism upon removal [171]. It is crucial to avoid agglomeration and allow for better process control.

In hyperthermia, the higher the intensity of the applied AC field, the higher the SAR produced. However, a ‘safe’ magnetic field for humans is considered to be H × f < 5 × 10^9^ A^−1^ m^−1^ s^−1^ [172, 173]. Various studies [170, 172] have investigated how to optimize the use of MNPs by particle size, shape, material, anisotropies, applied fields, and other characteristics. In terms of size, larger particles have shown a higher SAR [174]. However, if the nanoparticle becomes too large, it becomes a detriment since Néel and Brownian relaxations become less efficient as the magnetic moment may not align with the crystal lattice at larger sizes.

**Figure 6.**
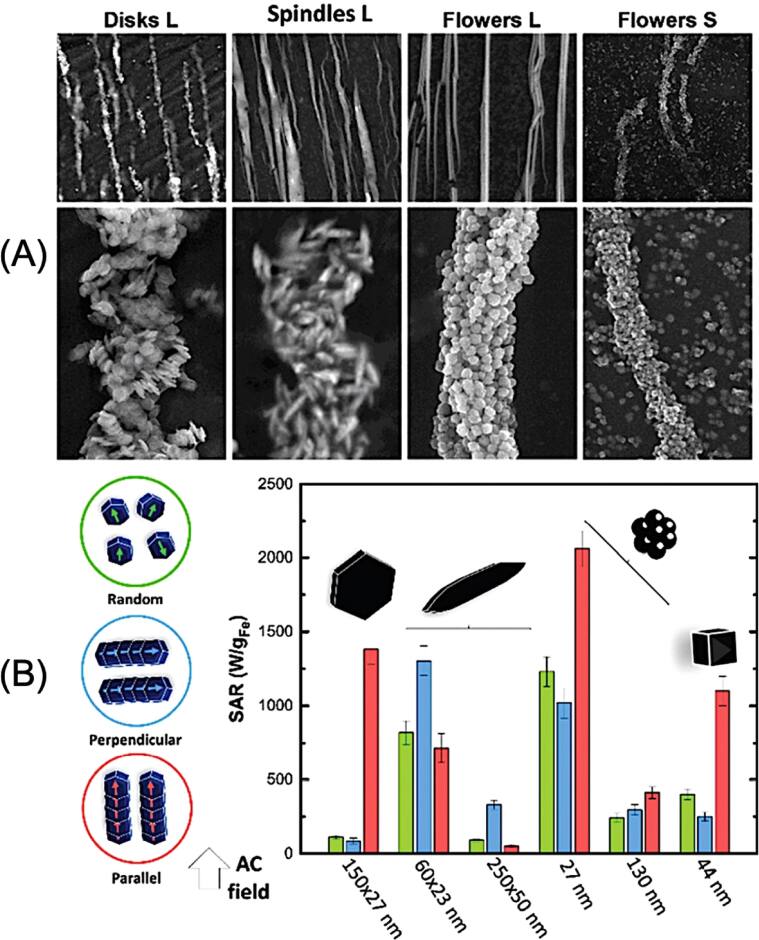
(A) Scanning electron microscopy images showing the effect of morphology on the organization of MPNs, including oriented large disks (L), large spindles (L), large flowers (L), and small nanoflowers (S). (B) SAR values of these particles vary as a function of size, shape, and AC magnetic field alignment direction. The MNPs were in agarose at a concentration of 2 mg ml^−1^. The strength of the AC magnetic field was 24 kA m^−1^, and the frequency was 765 kHz. Figure reprinted with permission from [172], copyright 2021 The Royal Society of Chemistry, licensed under CC BY-NC 3.0.

Gavilan *et al* [172] reported different MNP shapes such as spindles (i.e., elongated nanoparticles), cubes, flowers, and disks, as well as their effectiveness dependent upon size, as shown in figure 6(A). These authors found that the shape of the MNP is of particular interest due to the different shape anisotropies that alter the magnetic properties of the particles. Depending on the shape, which changes the shape anisotropy, the internal magnetic moment may have a preference regarding orientation. This can heavily impact the effectiveness of hyperthermia, as the performance may depend on what direction the applied external AC magnetic field is aligned with the nanoparticle. For example, figure 6(B) presents the preferred directional alignment for various particle shapes, which was observed to be either parallel, perpendicular, or random. The preferred direction of the externally applied field would be congruent with the bar that has the highest SAR, correlating by color for alignment. Some different sizes for spindles and flowers are also included to show how the effectiveness of SAR differs. Additionally, another important factor is how the MNPs interact and arrange together, which depends on the media that the MNPs are within, the MNPs’ intrinsic characteristics, the particle concentration, and the applied AC field. These authors also found that nanoflowers and spindles were the most efficient shapes for hyperthermia applications. The SAR value for 24 nm nanoflowers was around 500 W g^−1^ under H × f = 4.4 × 10^9^ A^−1^ m^−1^ s^−1^ and for 87 × 16 nm spindles was 400 W g^−1^ under H × f = 4.8 × 10^9^ A^−1^ m^−1^ s^−1^.

Vicentini *et al* [173] investigated a critical aspect of magnetic hyperthermia: the influence of the applied magnetic field. Their study considered the effects of coil geometry and positioning relative to the body. Optimizing the coil setup to minimize current requirements is essential for avoiding technical challenges in high-current environments. Using a mouse model, the researchers evaluated three coil types, Helmholtz, helical, and pancake, in *in vivo* hyperthermia experiments. They found that coils positioned farther from the body generated weaker magnetic fields and had a reduced therapeutic effect. In contrast, configurations that surrounded or encapsulated the body delivered stronger and more uniform fields across the entire subject. For testing, they used citrate-coated iron oxide MNPs at a concentration of 1.25 mg cm^−3^. Among the configurations, the helical coil that surrounded the animal proved most effective, offering both high temperature increases and efficient field generation without requiring high currents. Importantly, the applied field adhered to safety limits (H × f < 5 × 10^9^ A^−1^ m^−1^ s^−1^), and higher frequencies consistently led to greater temperature elevations. The researchers concluded that the helical coil setup was optimal, combining effective heating performance with minimal invasiveness and current demand.

Nowadays, there are many challenges associated with magnetic hyperthermia, which are the focus of the scientific community’s efforts. For instance, in a variety of systems, a high concentration of MNPs is often needed for the treatment to be effective [175]. This creates a potential issue regarding the long-term toxicity of the nanomaterials employed, which is yet to be studied. Additionally, effective heating using MNPs can be difficult to achieve, depending on whether the MNPs are well dispersed in the medium, or if the AC magnetic field is in the right direction or at the right strength [176, 177]. Thus, overcoming these present challenges is of paramount importance for this promising technology to develop.

### 3.2. MNPs for drug and gene delivery

Drug and gene-targeted delivery represent another cancer treatment approach that has been gaining rapid attention in recent years. However, there are various issues associated with this technology, particularly those related to cell penetration as well as degradation of the delivery components. In this regard, MNPs have been introduced as a novel vehicle to enhance drug and gene therapy performance due to their structural malleability, which allows a variety of chemical compositions, sizes, shapes, and surface charges [178], in addition to their enhanced biocompatibility [179], low toxicity and side effects [179, 180], and superparamagnetic behavior. Release conditions can be triggered by different stimuli, such as changes in the pH [181], exposure to magnetic fields [175], or temperature variations [182]. Besides cancer, gene therapy can be used to treat genetic disorders [178], which highlights the need for optimized delivery platforms.

**Figure 7.**
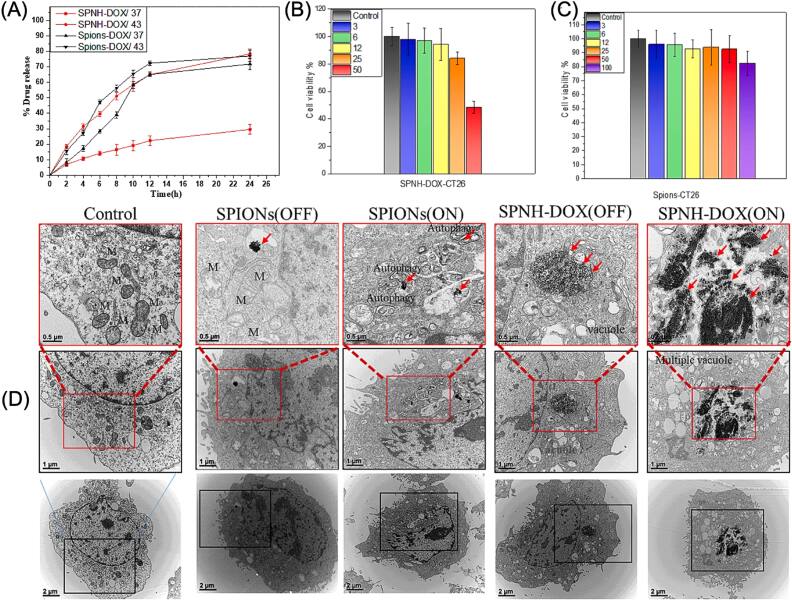
*In vitro* therapeutic evaluation of SPNH-DOX and SPION-DOX nanocarriers. (A) *In vitro* DOX release percentage over 24 h at 37 °C and 43 °C, where SPIONs-DOX represents the SPIONs directly loaded with DOX. (B) Different concentrations (3: blue, 6: green, 12: yellow, 25: orange, and 50: red μg/ml) of SPNH-DOX and the results of their biocompatibility, with studies presenting CT26 cell viability percentage. All the concentrations are compared to a control. (C) Different concentrations (3: blue, 6: green, 12: yellow, 25: orange, 50: red, and 100: purple μg/ml) of SPIONs and their CT26 cell viability percentage. All the concentrations are compared to a control. (D) TEM images of CT26 cancer cells after 24 h of exposure to different MNPs. Areas highlighted in red are meant to show where the photo above is focused, and red arrows indicate vacuoles and autophagy in the CT26 cells. The notation (ON) and (OFF) refer to the use of an AMF for treatment or the lack thereof, respectively. The bottom row shows the nanoparticle-treated CT26 cells. Figure reprinted with permission from [182], copyright 2023 The authors, published by Elsevier Ltd, licensed under CC BY-NC-ND 4.0.

Thermosensitive nanocarriers have been recently reported for drug delivery applications. For instance, Rezk *et al* [182] employed SPIONs coated with poly(N-isopropylacrylamide)/N (hydroxyethyl) acrylamide and loaded with the anti-cancer drug doxorubicin (DOX), labeled as SPNH-DOX. The coating had temperature-responsive properties, staying hydrated with blocked pores at temperatures below 41 °C. Above 41 °C, the polymer could collapse, releasing DOX. The authors included hyperthermia to heat the SPIONs, using an AC magnetic field with a strength of 10.06 kA m^−1^ and a frequency of 293 kHz. The *in vitro* drug release test is shown in figure 7(A), where at 24 h, the DOX released from the SPNH at 43 °C was 78.4%, and at 37 °C, this value was only 29.5%. The biocompatibility and cytotoxicity of the nanomaterial were also examined, and it was determined that the CT26 cells showed 50% viability at 50 μg ml^−1^ of SPNH-DOX, as presented in figure 7(B). The SPIONs were found to have excellent biocompatibility, as presented in figure 7(C). The use of this engineered ‘smart polymer’ shows very promising results when observing their interaction with cancer cells in figure 7(D). The combinatorial factor of using both hyperthermia and chemotherapy seems to have the greatest effect on the tumor-cultured cells. In figure 7(D), it is shown how the material negatively affected the growth of cancer cells in comparison to non-cancerous cells.

**Figure 8.**
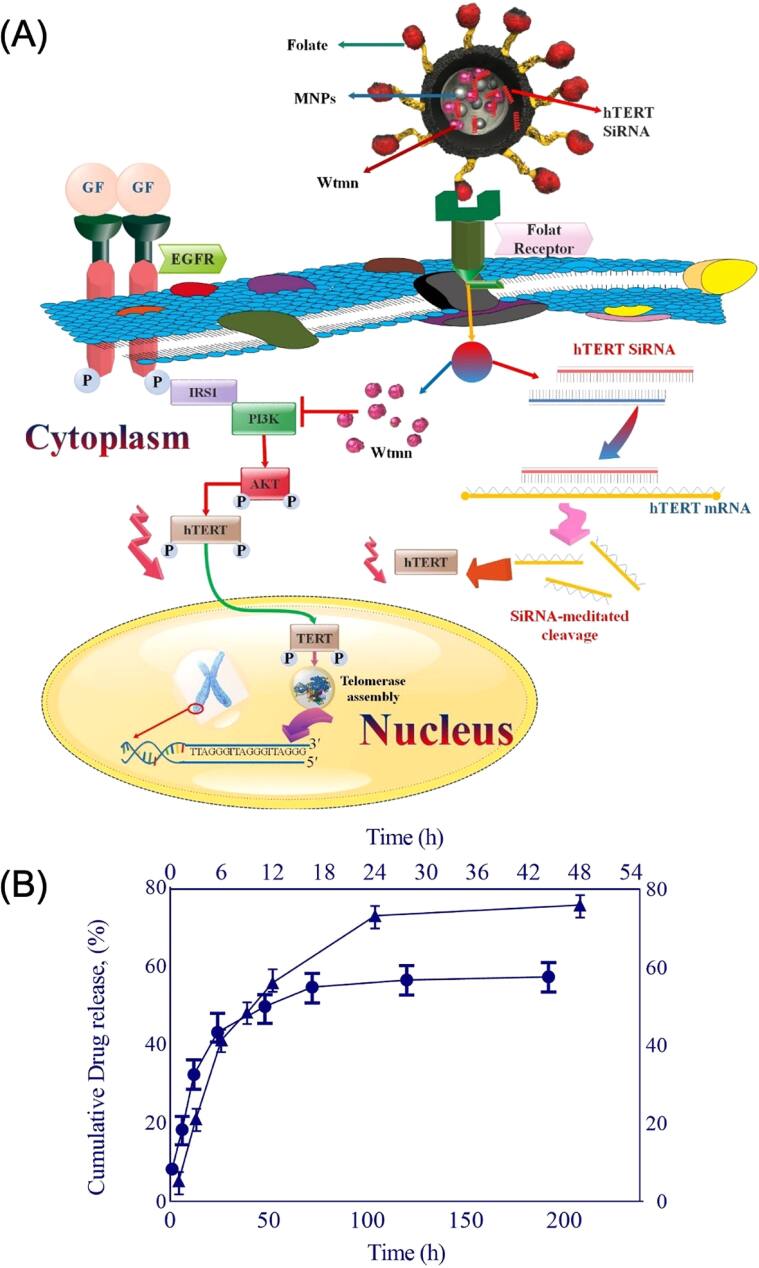
(A) A holistic look at the targeting and delivery process involved in the study reported by Ghareghomi *et al*, where the MNPs composed of PLGA functionalized with folic acid (MNP-PLGA-FA) were co-loaded with hTERT siRNA and Wtmn, which worked to suppress hTERT mRNA, as well as PI3K and AKT/mTOR signaling pathways. This suppressed hTERT and inhibited telomerase repair. (B) Wtmn (triangles) and siRNA (circles) *in vitro* release from the Wtmn and siRNA-loaded MNP-PLGA-FA over 48 h and 8 days, respectively. Figure reprinted with permission from [183], copyright 2021 Elsevier Inc.

Gene therapies for cancer treatment are meant to suppress the effect of various genes that facilitate cancer cell growth (oncogenes). Often, this requires special conditions to achieve the best outcomes, and thus, MNPs are ideal for this purpose due to their chemically adaptable surface. One of the most targeted genes is human telomerase reverse transcriptase (hTERT), which maintains telomeres and enables cells to divide. In cancer, dysregulation of hTERT causes the cells to divide infinitely, thus allowing for accelerated cell growth. Various studies have focused on silencing this gene using MNPs, such as the one performed by Ghareghomi *et al* [183], who targeted hTERT using siRNA (small interfering RNA), wortmannin (Wtmn, a PI3K and AKT/mTOR signal pathway inhibitor), and MNPs to ensure accurate delivery in ovarian cancer. MNPs were encapsulated with folic acid, as folate receptors are often overexpressed, especially in ovarian cancer. The authors observed that the combination of different drugs proved effective, as when considering their mechanisms carefully, they amplified each other in the *in vitro* models. The release mechanism was the slow breakdown of the PLGA shell and passive diffusion. Over 48 h, 75% of Wtmn was released, and over 8 days, 58% of siRNA was released, as presented in figure 8(B). The structure of the nanomaterial and the schematics of the release process can be observed in figure 8(A).

**Figure 9.**
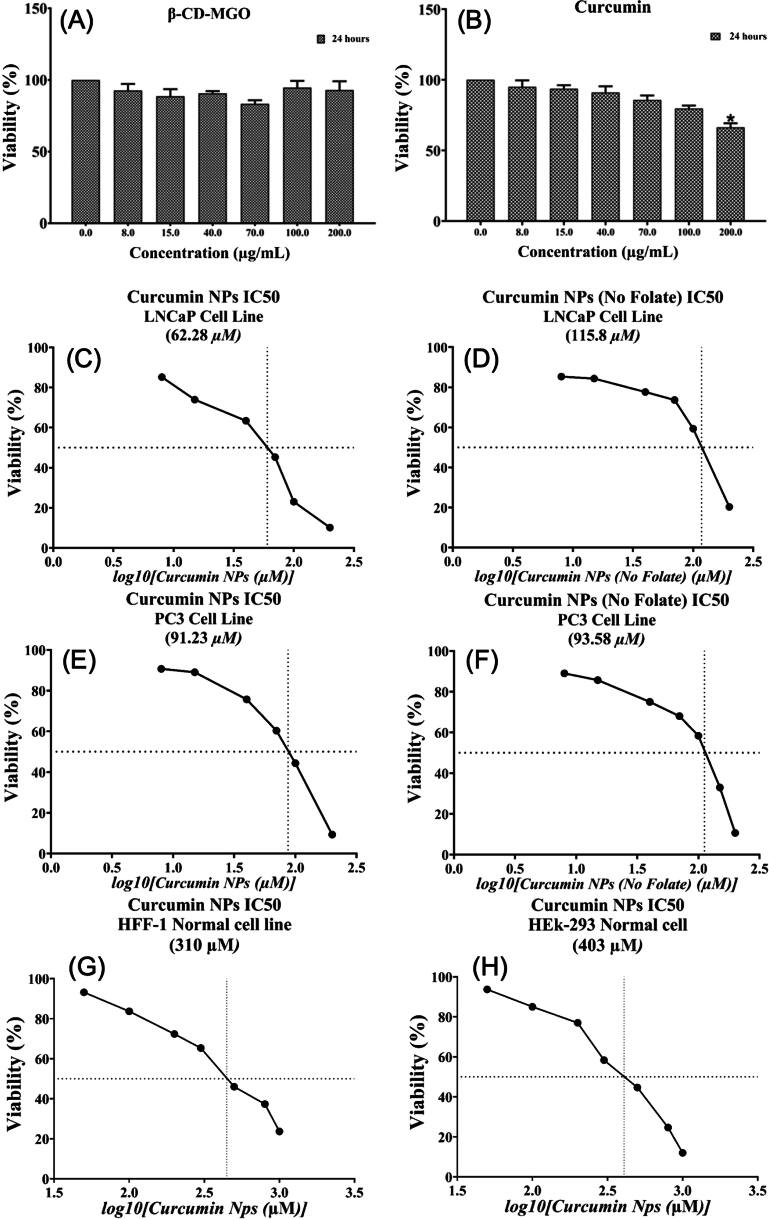
Cytotoxicity and biocompatibility test results towards the cultured LNCaP prostate cancer cells after 24-hour incubation at different material concentrations for (A) *β*-CD-MGO, and (B) curcumin (*P < 0.05). Evaluation of the material performance (with and without folate functionalization) against various cancer cell lines: (C) curcumin@*β*-CD-MGO and (D) folic acid-curcumin@*β*-CD-MGO incubated with LNCaP cells for 24 h, highlighting the effect of folate functionalization on IC_50_ values; (E) Viability studies of curcumin@*β*-CD-MGO and (F) folic acid-curcumin@*β*-CD-MGO incubated with PC3 cells after 24 h. Line graphs showing cytotoxicity of two healthy human cell lines: (G) HFF-1 and (H) Hek-293, confirming reduced toxicity compared to cancer cells. Figure reprinted with permission from [184], copyright 2024 Elsevier Ltd.

The use of folic acid as a coating for MNPs was also exploited by Einafshar *et al* [184], who employed magnetic graphene oxide (MGO) loaded with curcumin to inhibit cancer cell growth. Curcumin does not have good biocompatibility and thus, it was proposed to be used as the cargo of MGOs. *β*-cyclodextrin (*β*-CD) was also incorporated into the nanomaterial for its hydrophobic inner cavity, where the equally hydrophobic curcumin could be stored. Folic acid coatings were utilized to target the tumor cells. They evaluated the biocompatibility of various materials, including *β*-CD-MGO (figure 9(A)) and curcumin (figure 9(B)), and did not notice substantial effects when they were employed separately. Cytotoxicity studies against various cancer cell lines (figures 9(C)–(F)) were also performed. From the results with LNCaP cells, it can be observed that, according to their half-maximal inhibitory concentration (IC 50) values, the folic acid-curcumin@*β*-CD-MGO was more effective for cancer treatment, with only 62.28 μM IC 50 compared to the less efficient curcumin-*β*-CD-MGO, the material without folate functionalization, which reported a much greater IC 50 value (i.e., 115.8 μM). Figures 9(E) and (F) shows the evaluation of the material with the PC3 cell line, which is a human prostate cancer cell line, and a similar difference between the material functionalized with and without folate was observed. The cytotoxicity towards healthy human cells was tested, which is shown in figures 9(G) and (H), and the measured IC 50 concentration was higher than the value reported for cancer cells, showing minimal cytotoxicity of the prepared folic acid-curcumin@*β*-CD-MGO, highlighting the promising role of this material in future cancer therapies.

Despite the promising applications of MNPs for drug and gene delivery, the area faces several challenges that hinder translation into clinical use, such as uncertainties regarding patient comfort and potential side effects [179]. Additionally, further studies need to ensure that the materials are effective against all the different extant cancer cell lines, as well as the different types of tumors and their microenvironments. Performing these studies might be key to the future implementation of these technologies.

### 3.3. MNPs for combination therapies

Different approaches are being pursued by the scientific community to employ hybrid MNP materials in dual therapies against cancer. Often, these materials and their combined modalities are more effective than using single therapies separately. The special properties of MNPs, including their ability for drug and gene transport, their biocompatibility, their superparamagnetism, etc, make them promising materials for the development of novel theragnostic platforms for cancer diagnosis and treatment [185, 186]. For instance, as presented above, hyperthermia can be used in combination with drug and gene therapy, using thermosensitive nanocarriers releasing their loaded drugs (i.e., doxorubicin) through a change in temperature using MNP hyperthermia [182]. Liu *et al* [187] provided a material targeting liver cancer. This material involved CD20, the heat shock protein inhibitor (HSPI), magnetite, and silica-based nanoparticles (CD20-HSPI&FE_3_O_4_@SiNPs) that had dual functionality of hyperthermia and chemotherapy treatment. Chemotherapy was achieved through the release of HSPI, which inhibited the ability of cancer cells to repair themselves after heat-induced damage. This worked synergistically with hyperthermia, resulting in an effective form of combination therapy.

**Figure 10.**
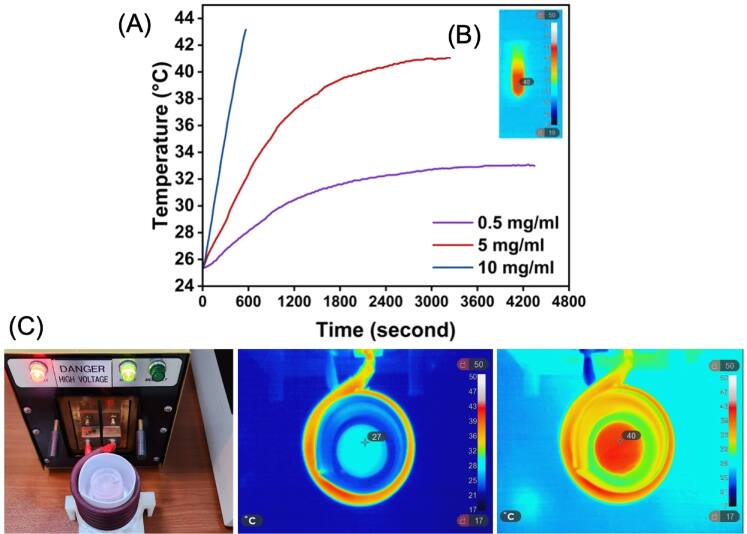
(A) Hyperthermia measurements for magnetic silica nanoparticles under an AC magnetic field of 1.59 × 10^6^ A m^−1^ and 250 kHz. (B) A thermal photograph was taken as the last temperature recorded for the 10 mg ml^−1^ sample, showing that the solution was kept at 43 °C. (C) Photos of a magnetic hyperthermia coil device (left), a thermal image of the room temperature sample solution before inducing hyperthermia (middle), and a thermal photo of the solution heated using magnetic hyperthermia (right). Figure reprinted with permission from [188], copyright 2024, The Author(s), under exclusive license to Springer Science Business Media, LLC, part of Springer Nature.

In a different study [188], MNPs loaded with a gene (i.e., pHSP-Azu plasmid, designed to encode anti-cancer azurin) could be released via heat. This encoded anti-cancer azurin was meant to be internalized by the target cells after crossing the cellular membrane to alter the inner functionalities of the cell. The modality by which heat was achieved in this study was magnetic hyperthermia. Magnetic fields of 1.59 × 10^6^ A m^−1^ were applied to an 11.6 mg sample of the nanomaterial, and the increase in temperature is presented in figures 10(A) and (C), where the nanomaterial achieved 43 °C for around 8 min at a frequency of 250 kHz. A thermal image (figure 10(B)) confirmed the capabilities of this material. Not only would the cancer cells be damaged by hyperthermia, but also by the release of Azu, the gene encoder for azurin, to ensure a more effective treatment. Moreover, the authors exploited ferroptosis, cell death via a lethal amount of iron exposure, to stop cancer cell growth.

**Figure 11.**
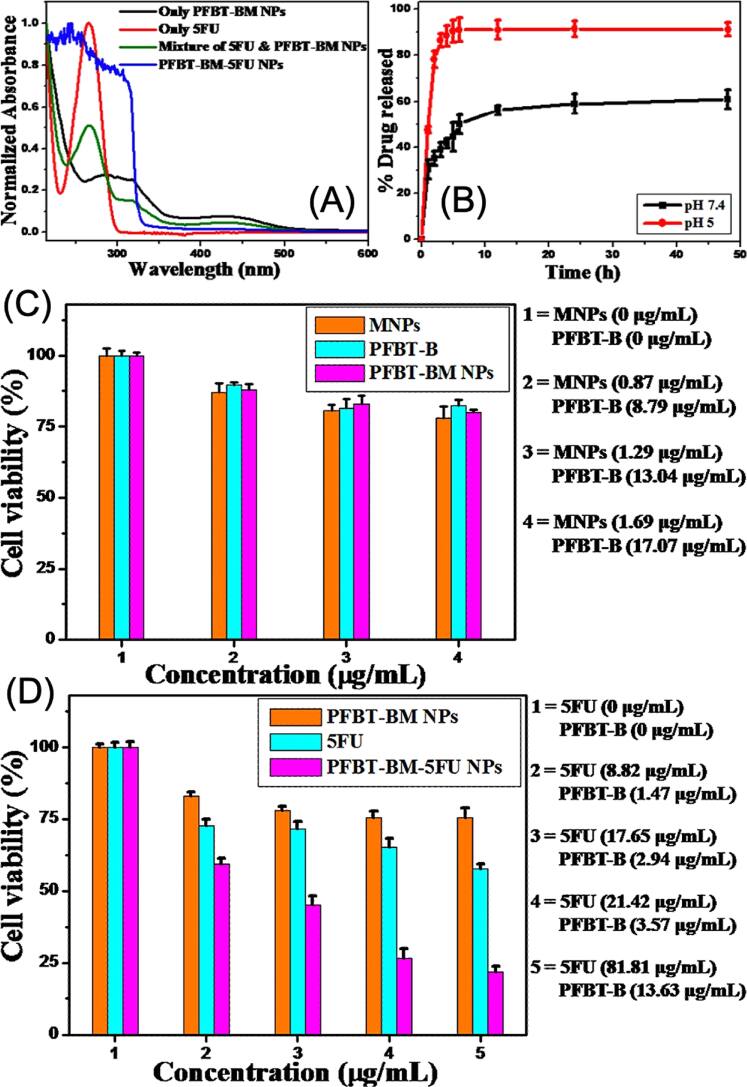
(A) Fluorescence spectra of different materials: PFBT-BM NPs, 5FU, a mixture of 5FU and PFBT-BM NPs, and PFBT-BM-5FU NPs. (B) Percentage of 5FU released from PFBT-BM-5FU NPs at a pH of 5 and 7.4. (C) Cytotoxicity of MNPs, PFBT-B, and PFBT-BM NPS on HEK 293T cells at differing concentrations. (D) Cytotoxicity of PFBT-BM NPs, 5FU, and PFBT-BM-5FU NPs on cancer HeLa cells. Figure reprinted with permission from [189], copyright 2020 Elsevier Ltd.

The research from Kalita *et al* [189] detailed a promising theranostic approach in which 5-Fluorouracil (5FU), an anti-cancer drug, was loaded onto a polymer biomolecule (PFBT-B) coated on MNPs. Their approach combined imaging as well as drug delivery. PFBT-B served as a non-toxic fluorescent coating with the ability to target malignant tumors. The fluorescent effectiveness of this coating is shown in figure 11(A). The tumor-targeting capability of the PFBT-B-coated MNPs (PFBT-BM NPs) was also assessed, and it was found that the particles showed higher internalization in HeLa cancer cells than in healthy HEK 293T cells, a human embryonic kidney cell line. The *in vitro* drug release mechanism was found to be a change in pH, as tumor cells often present a lower pH in their microenvironment than normal cells. Figure 11(B) presents the drug release results at low pH (i.e., 5, mimicking that of the tumor microenvironment), as well as the release rate at a pH of 7.4 (i.e., mimicking the pH of plasma). The drug release results demonstrated the ability of the material to deliver the 5FU only at the pH encountered in the tumor microenvironment. Biocompatibility studies were performed with healthy human cells (i.e., HEK 293T), and the results (figure 11(C)) showed good cell viability even at high concentrations of the material. Finally, figure 11(D) shows the cytotoxic properties of the MNPs against cancer HeLa cells as a function of material (5FU and PFBT-B) concentration, which demonstrates how cell viability decreased for the hybrid material. Overall, the combination of drug delivery and fluorescence imaging provides the advantage of both diagnosis and treatment, which, as proposed by these authors, has great potential to be used in cancer treatment.

**Figure 12.**
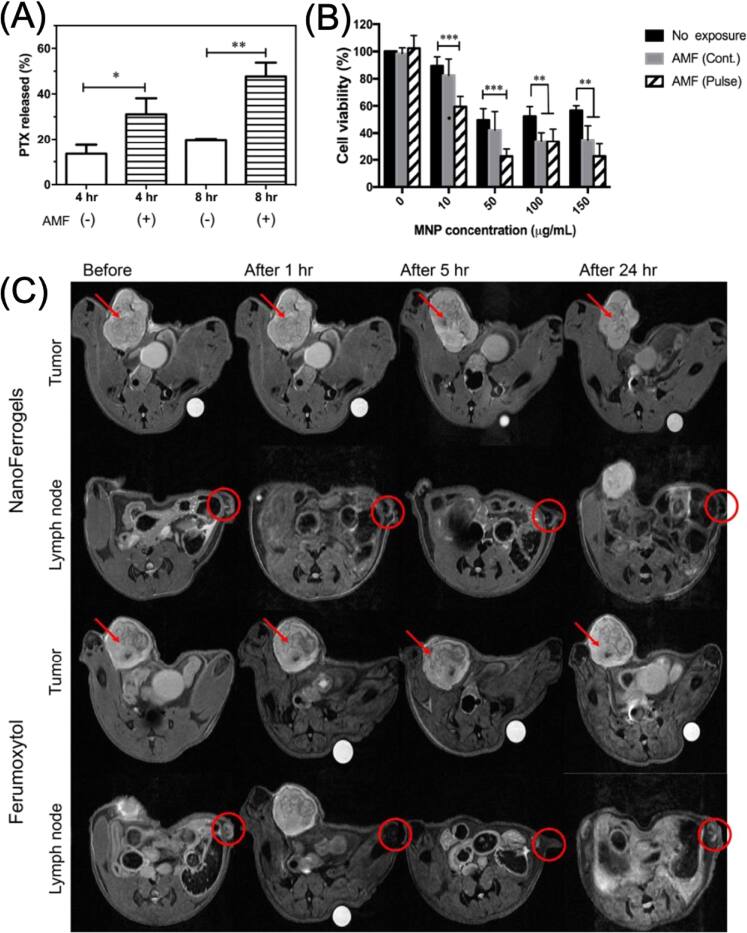
(A) 4- and 8-hour PTX release, with (shaded bars) and without (white bars) an AC magnetic field. (B) Cytotoxicity studies using LCC-6-WT cancer cells incubated with the material at different concentrations and conditions, including no AC magnetic field, a pulsed field, and a continuous AC magnetic field. (C) T_2_-weighted images of tumors using both NFG and Ferumoxytol before and after exposure, and after 1, 5, and 24 h. The arrows point at tumors, and the circles encapsulate the lymph nodes. Figure reprinted with permission from [190], copyright 2021 Elsevier Inc.

Seo *et al* investigated a novel MNP material and its usage for MRI, drug delivery, and remote control (using magnetic fields) [190]. The authors used NanoFerrogels (NFGs), comprising MNPs that worked as imaging contrasts and poly(2-oxazoline) (pOX) based polymeric micelles that had a large drug loading capacity as well as high biocompatibility. Paclitaxel (PTX) was the chemotherapeutic drug used, as it could be loaded onto pOX at a high capacity of up to 45% w/w [191]. The MNPs were anchored to the surface of the micelles using dopamine-functionalized pOX micelles. The resultant large drug capacity, combined with the inherent properties of MNP, allowed them to use the material for both imaging and drug delivery. Indeed, they observed increased drug release with the use of a low-frequency AC magnetic field (50 kA m^−1^, 50 Hz). Due to this low frequency, the MNPs did not rotate fast enough to generate any heat. However, they experienced mechanical stress acting on the pOX-PTX micelle, causing it to break and release the PTX at much higher rates than without the MNPs’ induced rotational mechanisms (figure 12(A)). The effectiveness of *in vitro* PTX release from the NFGs is displayed in figure 12(B), where a pulse AC magnetic field was utilized, resulting in the best cancer cell destruction property. The pulses consisted of 10 min on and five minutes off routines for a total of 30 min. These MNPs demonstrated an enhanced MRI imaging capability, as shown in figure 12(C), with scans performed *in vivo*. The scans showed that the NFGs had better signal integrity than the commercially available contrast agent Ferumoxytol. The darker areas in figure 12(C) are representative of good signal integrity, and the tumor steadily became darker over 24 h, showing that the contrast was available for up to 24 h. Overall, this work presents a theranostic NFG platform that has the potential to be used for drug delivery and MRI dual treatment-imaging.

MNPs with dual capabilities are an exciting prospect for cancer treatment. Developing multimodal systems able to not only treat but also diagnose is of great interest. However, several challenges need to be overcome, such as cellular uptake [184, 189, 190], which is an issue that needs attention in order to develop effective therapies [184]. Aggregation is also a problem commonly discussed in the literature [184, 190]. This can affect the material’s intended functions, but can also be overcome by different surface coatings.

## 4. Applications in cancer diagnostics

### 4.1. Magnetic Resonance Imaging (MRI)

#### 4.1.1. MRI working principle and MNPs as contrast enhancers

**Figure 13.**
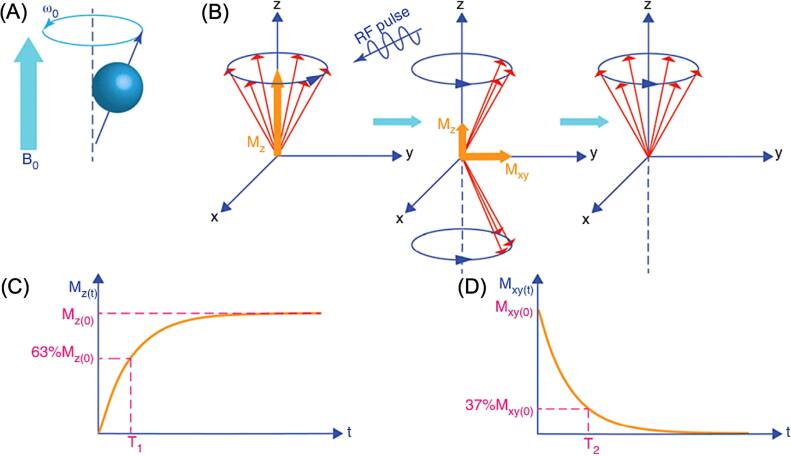
Schematic representation of the working principle of MRI. (A) Under the influence of an external magnetic field (B_0_), protons align in either the parallel (majority) or antiparallel (minority) orientation and begin to precess around the magnetic field axis at the Larmor frequency ${\omega }_{0}$. (B) When RF pulses are applied at the Larmor frequency, protons absorb energy, and the net magnetization is tipped away from the longitudinal axis. After the RF pulse is turned off, the system begins to relax back to equilibrium. (C) T_1_ relaxation is the time taken to attain 63% of the initial longitudinal magnetization M_z_. (D) T_2_ relaxation or spin–spin relaxation, in which protons begin to dephase, resulting in a decrease of the transverse magnetization M_xy_. Figure reprinted with permission from [192], copyright 2016 Wiley Periodicals, Inc.

Known for its ability to produce high-resolution images of soft tissues without ionizing radiation, MRI is one of the most widely utilized diagnostic tools in medicine. MRI provides critical insights into anatomical and physiological conditions, particularly in brain imaging, tumor detection, and cardiovascular assessments. The fundamental principle of MRI revolves around the detection of radiofrequency (RF) signals emitted by precessing proton spins after excitation in a strong external magnetic field (B_0_). When placed in B_0_, hydrogen nuclei align either in parallel (low energy) or antiparallel (high energy) to the field, with a slight excess in the parallel state, generating a net magnetization (M_z_) along B_0_, as presented in figure 13(A). When an RF pulse is applied, the spins absorb energy and flip into the higher energy state. This also causes the spins to precess in phase, generating a transverse magnetization (M_xy_) perpendicular to B_0_ (figure 13(B)). When the RF pulse is removed, the excited spins return to equilibrium through two simultaneous processes (figures 13(C) and (D)): T_1_ relaxation (longitudinal relaxation), during which spins exchange energy with the surrounding lattice, restoring the longitudinal magnetization M_z_; and T_2_ relaxation (transverse relaxation), which occurs due to spin–spin interactions, leading to dephasing of M_xy_. The precessing M_xy_ induces a voltage in receiver coils, which is detected as the MRI signal. Then, spatial encoding gradients are applied to localize the signal origin, allowing the reconstruction of a 3D image.

The contrast in MRI images arises from differences in T_1_ and T_2_ relaxation times in various tissues. The inherent low sensitivity of MRI requires the use of contrast agents to enhance the image quality by modifying proton relaxation times. In this regard, magnetic nanomaterials, and particularly SPIONs, have emerged as highly effective T_2_-weighted contrast agents [193]. The benefits of using SPIONs stem from their ability to generate local magnetic field inhomogeneities, which can enhance susceptibility differences and accelerate the dephasing of nearby protons. This shortens the T_2_ relaxation time, leading to a signal reduction in T_2_-weighted MRI images, particularly in areas where SPIONs accumulate. In addition to SPIONs, gadolinium (Gd^3+^)-based nanoparticles have also been demonstrated to be potent T_1_ contrast agents, providing bright (positive) contrast in MRI images. This is due to Gd^3+^ ions possessing seven unpaired electrons, resulting in a strong paramagnetic effect that shortens the longitudinal relaxation time of surrounding water protons [194]. The incorporation of these agents into nanoscale carriers, such as dendrimers, liposomes, and mesoporous silica, significantly enhances their relaxivity and biocompatibility while reducing potential toxicity [195–197].

Moreover, nanoparticles can contain a large fraction of magnetic material, amplifying the contrast effect at lower doses in comparison to bulk materials [198]. Due to their small size, their large surface area allows functionalization with targeting ligands or therapeutic agents at a high loading ratio. Also, their EPR effect enables them to selectively accumulate in tumor tissues, improving the signal-to-noise ratio (SNR). The efficacy of MNPs as MRI contrast agents is closely tied to their structural features, such as size, shape, crystal structure, and surface modification. Solomon-Bloembergen-Morgan (SBM) theory has been a guiding principle for understanding the relaxation enhancement effects of paramagnetic systems [199]. However, this classical model struggles to fully explain the relaxation mechanisms in modern, structurally complex, magnetic nanomaterials [200, 201]. Recent studies, like the one reported by Zhou *et al* [202], convey that size and shape significantly impact relaxivity. IONPs, for example, exhibit size-dependent T_2_ relaxivity, with larger particles providing stronger contrast due to greater perturbation of proton spin coherence. Nevertheless, by combining advanced imaging capabilities with potential therapeutic functionalities, MNPs have redefined the landscape of MRI contrast enhancement. Future advancements in their design and functionalization promise to further enhance their efficacy and safety, paving the way for next-generation diagnostic tools [203].

#### 4.1.2. MRI for tumor imaging

MNPs have been extensively utilized as MRI contrast agents for cancer imaging, demonstrating their versatility for enhancing imaging sensitivity and enabling targeted drug delivery. A study with doxorubicin-modified SPIONs was reported to enhance T_2_-weighted MRI signals and facilitate tumor-targeted chemotherapy monitoring through magnetic field guidance [204]. In another study, SPIONs conjugated with folate showed superior MRI sensitivity and selective drug delivery for treating liver cancers, specifically by targeting folate receptor-expressing tumors [205]. The use of multi-layer *β*-cyclodextrin and Pluronic^®^ F-127 (PF-127) polymer-coated MNPs (F127250) recently manifested a high T_2_ relaxivity and improved MRI contrast, particularly when loaded with curcumin, highlighting their potential for theranostic applications [206]. Additionally, human epidermal growth factor receptor 2 (HER2)-targeting bacterial magnetosomes have been recently developed to significantly enhance MRI sensitivity and specificity for HER2-positive breast cancer detection, showcasing advancements in tumor-targeting imaging strategies [207]. These studies support the idea that MRI serves not only as an advanced tool for tumor imaging but also as a platform for guided therapy delivery. MRI has also been exploited for enhanced tumor classification, improving diagnostic accuracy, as exemplified in various studies utilizing an MRI-based approach for brain tumor classification [208] and dual-modality MRI-photoacoustic imaging for renal fibrosis detection [209].

**Figure 14.**
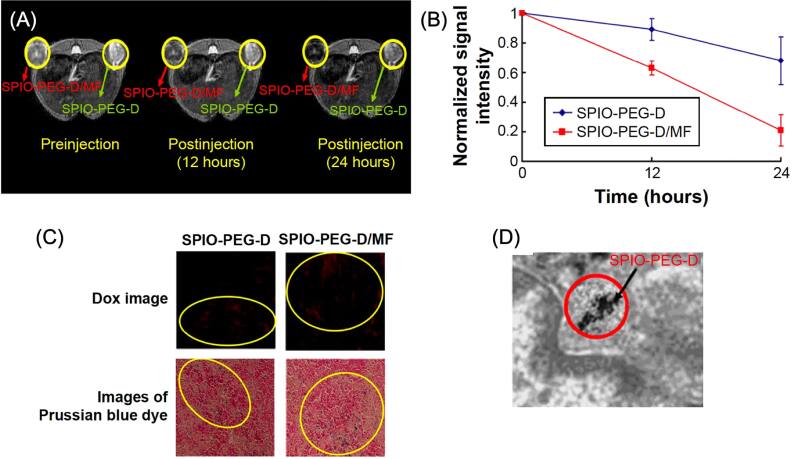
*In vivo* MRI images and detection of SPIO-PEG-D under a magnetic field for tumor targeting. (A) T_2_-weighted MRI images of tumor tissues before and after SPIO-PEG-D treatment. (B) Normalized MRI signal intensity in mouse tumors with SPIO-PEG-D and magnetic field-assisted treatment (SPIO-PEG-D/MF). (C) Confocal laser-scanned microscopy images of doxorubicin in tumors and Prussian blue-stained microscopy images of iron deposits in tumor tissues 24 h post-treatment with SPIO-PEG-D and SPIO-PEG-D/MF. (D) TEM images depicting SPIO-PEG-D deposition in tumor cells. Figure reprinted with permission from [204], copyright 2016 Taylor & Francis.

Additionally, MRI has played a critical role in therapy-guided delivery, including the optimization of gold-layered multifunctional nanoparticles for enhanced proton therapy [210] and hollow manganese-doped calcium phosphate functionalized particles for photothermal and anti-angiogenic cancer therapy [91]. For instance, Lin *et al* reported the development of doxorubicin-conjugated SPIONs coated with polyethylene glycol (SPIO-PEG-D) for MRI monitoring and enhanced tumor chemotherapy [204]. Their material leveraged a magnetic field to achieve a high intratumoral accumulation, as revealed by Prussian blue staining. Their results emphasized the dual benefits of MRI imaging and targeted chemotherapy enabled by SPIO-PEG-D. The efficiency assessment of SPIO-PEG-D in tumor targeting and drug retention under a magnetic field is shown in figure 14, where changes in T_2_-weighted MRI signal intensity before and after treatment are illustrated in figures 14(A) and (B), emphasizing enhanced retention with magnetic assistance. Confocal microscopy and Prussian blue-stained images of the tumor tissue showed the increased iron deposition and doxorubicin presence in the magnet-assisted group (figure 14(C)). TEM images, shown in figure 14(D), further confirmed the localization of nanoparticles within tumor tissues, reinforcing their role in targeted therapy.

Additionally, Zhang *et al* developed a novel MRI contrast agent aiming to enhance the transverse relaxation rate for a better contrast in T_2_-weighted MRI images [207]. After obtaining magnetosomes from Magnetospirillum gryphiswaldense MSR-1 bacteria, the authors modified them with an anti-HER2 affibody. The synthesis involved removing the original membrane proteins from the magnetosomes and replacing them with a fusion protein consisting of the MamC anchor protein and an anti-HER2 affibody. The resulting nanoparticles, called BMW-HAF, were reported to exhibit a high transverse relaxivity (r_2_) value of 599.74/mM/s, good biocompatibility, and the ability to selectively target HER2-expressing breast cancer cells both *in vitro* and *in vivo*. When used as an MRI contrast agent, BMW-HAF significantly enhanced T_2_-weighted imaging of orthotopic breast tumors, especially those overexpressing HER2, with detectable signals from 3 to 24 h after administration. These findings collectively underscore the widespread application of MNPs as MRI contrast agents in tumor imaging and therapy, highlighting their multifaceted role in precise tumor detection and guided therapeutic intervention, paving the way for improved diagnostic and treatment capabilities.

### 4.2. Magnetic Particle Imaging (MPI)

#### 4.2.1. MPI working principle and MPI tracers

MPI is a technique that produces background-free images by leveraging the nonlinear magnetization responses of SPIONs under an oscillating magnetic field [25]. A strong selection field is applied to create a field-free point (FFP) or field-free line (FFL) regions where the magnetic field is effectively zero. Only SPIONs located in this zero-field region remain unsaturated and respond dynamically to the drive field, generating a time-varying magnetization signal rich in higher harmonics. In contrast, SPIONs outside the FFP/FFL are magnetically saturated and do not contribute to signal generation. The induced signal is detected by a receiver coil and reconstructed into images that map the spatial distribution of the tracers or signal-generating agents. In this case, the distribution of SPIONs is detected to construct the images. Since biological tissues do not produce MPI signals, this technique offers excellent contrast with zero background interference, making it highly suitable for *in vivo* imaging applications [211, 212].

**Figure 15.**
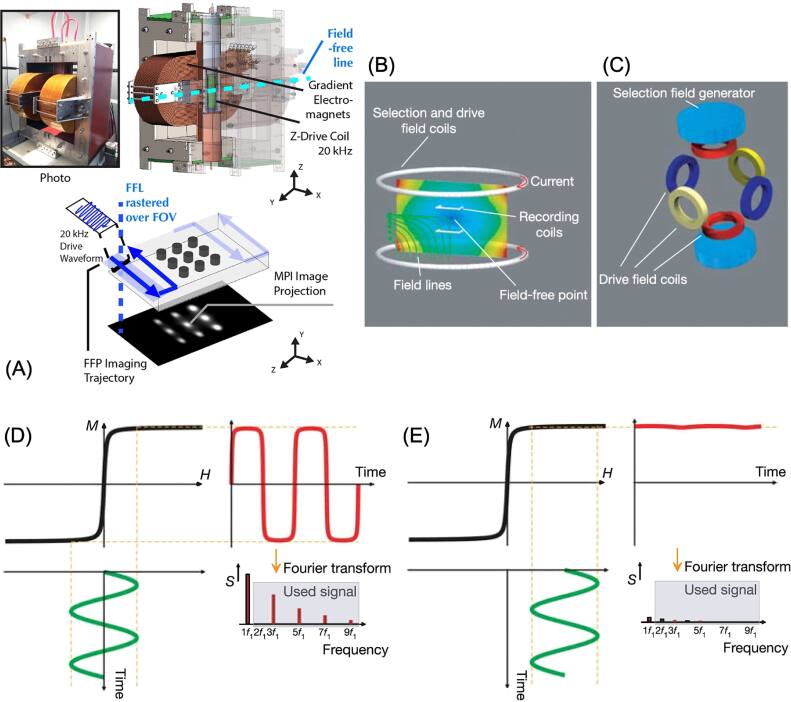
Schematic representation of the hardware setup and signal generation in MPI. (A) Berkeley MPI scanner setup showing image acquisition from the MNP interaction with an oscillating drive field. (B) The selection and drive field coils that establish an FFP for spatial encoding. (C) Drive field-based MPI configuration, enabling scanning flexibility with dynamic FFP control. (D) Nonlinear magnetization response of MNPs under a time-varying magnetic field that produces harmonic components used for MPI signal reconstruction. (E) MNP saturation response to a static magnetic field suppresses harmonic generation, leading to no usable signal for MPI image reconstruction. (A) reprinted with permission from [213], copyright 2018 American Chemical Society. (B)-(E) reprinted with permission from [214], copyright 2005 Macmillan Magazines Ltd.

Figure 15 provides an overview of the structural foundation and signal generation principles of MPI, illustrating the essential hardware components, magnetic field interactions, and particle response mechanisms required for MPI image reconstruction. The Berkeley MPI scanner (figure 15(A)) uses an FFL configuration for image acquisition. As the FFL moves, MNPs within this region respond to a 20 kHz drive field, producing harmonic signals used for image reconstruction. Figures 15(B) and (C) depict two complementary aspects of the same MPI system. Figure 15(B) highlights the selection field components - two large rings with opposing direct currents create a static magnetic gradient, forming FFP at the center. These rings also serve as drive field coils, superimposing alternating currents to manipulate the FFP position. Small harmonic recording coils detect MNP-induced signals. Figure 15(C) expands on this by showing the drive field encoding mechanism. Three pairs of opposing coils dynamically shift the FFP across the imaging volume, enabling spatial encoding without physical movement of the subject.

In figure 15(D), the nonlinear magnetization response of MNPs to an externally applied field is illustrated, with the oscillating magnetic field (green curve, H), as well as the resultant magnetization (black curve, M), producing a time-dependent signal (red curve) that generates higher harmonics critical for MPI image formation. The effect of a static magnetic field on MNP magnetization, as seen in figure 15(E), highlights that under a constant non-zero field, MNPs reach saturation, suppressing higher harmonics, thereby eliminating any effective image signal. The gray box highlights the absence of usable harmonics for MPI reconstruction from these saturated tracers.

The selection of suitable MPI tracers is critical to achieving optimal image quality. SPIONs, characterized by a Langevin magnetization behavior, serve as the primary tracers due to their unique magnetic properties, such as their characteristic saturation magnetization and relaxation dynamics. Current advances in MPI tracers focus on tailoring the magnetic core size and surface coatings to optimize SNR and spatial resolution [215]. Additionally, various IONPs and doped ferrite nanoparticles have been explored as potential MPI tracers as well, to enhance signal intensity and spatial resolution. Manganese ferrites (MnFe_2_O_4_) have been reported to exhibit shorter relaxation times and higher saturation magnetization than commercial tracers like Vivotrax, improving MPI performance [216].

Similarly, Zn- and Co-ferrite nanoparticles exhibit distinct dominant relaxation mechanisms due to their size- and anisotropy-dependent magnetic properties. Zn-ferrite (∼20 nm) balances Néel and Brownian relaxation for MPI performance comparable to Resovist, while larger Co-ferrite (∼30 nm) shows predominantly Brownian dynamics [217]. On the other hand, Co-doping has been demonstrated to enhance saturation magnetization and anisotropy, leading to stronger MPI signals and improved tracer performance, resulting in a two-fold increase in signal intensity compared to undoped tracers [218]. The development of superferromagnetic iron oxide nanoparticles (SFMIOs) that exhibit collective dipolar interactions has led to a ten-fold increase in MPI signal strength and spatial resolution compared to conventional single-core SPION tracers, offering potential clinical translation benefits while reducing hardware costs [219]. Therefore, optimizing the size, shape, composition, and surface coatings of MNPs plays a crucial role in enhancing MPI signal performance, for which advancements in MNP separation and characterization are of paramount importance for improved tracer development [220, 221]. Current research efforts are aligned to engineer MPI tracers for biomedical applications, with a focus on improving signal intensity, spatial resolution, and clinical feasibility.

**Figure 16.**
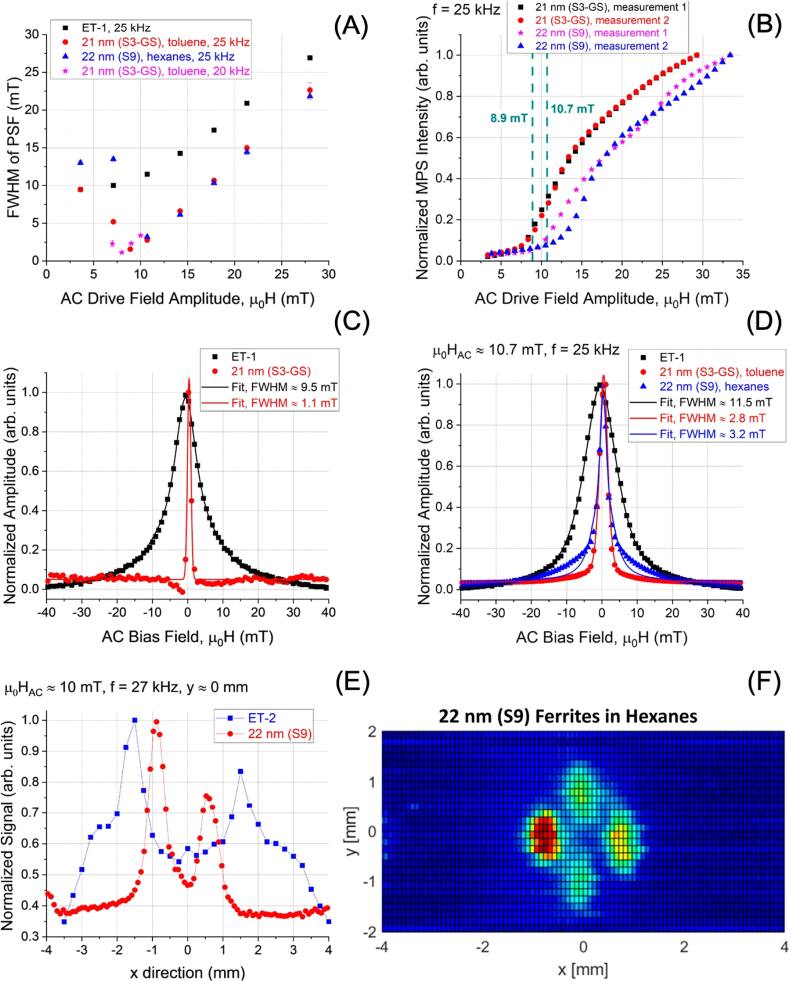
Comparison of strongly interacting ferrites to commercial MPI tracers and their impact on spatial resolution. (A) FWHM of the PSF as a function of AC drive field amplitude for 21 nm (S3-GS) and 22 nm (S9) particles compared to ET-1, a commercial tracer. (B) Normalized AC drive field amplitude dependence, showing a threshold effect corresponding to chain formation. (C) Sharpest PSF measured for 21 nm (S3-GS) ferrites and ET-1, demonstrating a 9× improvement in resolution with the synthesized material. (D) PSF comparison at high-field amplitudes, emphasizing the trade-off between signal enhancement and spatial resolution. (E) Normalized signal strength of MPI scans for 22 nm (S9) nanoferrites and a conventional tracer, ET-2, demonstrating enhanced resolution and signal intensity of the nanoferrites in resolving a phantom with a 2 mm separation. (F) 2D MPI image reconstructed using 22 nm S9 ferrite nanoparticles in a 4-channel phantom with approximately 2 mm separation in both x and y directions. The color mapping indicates signal strength, with darker colors representing higher concentrations of nanoparticles. Figure reprinted with permission from [222], copyright 2024 American Chemical Society.

The dipolar interactions between MNPs have also been exploited to enhance the performance of these materials in MPI applications. Abel *et al* investigated the importance of optimizing nanoparticle size, interparticle interactions, and drive field parameters to maximize MPI signal strength and resolution [222]. The work also validated how strongly interacting nanoferrites significantly enhanced MPI sensitivity and spatial resolution by forming particle chains through dipolar interactions, improving signal intensity up to 37 times and resolution up to 9 times in comparison to the commercially available MPI tracer Vivotrax. They synthesized ferrite magnetic particles in distinct sample batches with sizes ranging from 12 to 27 nm and investigated the influence of particle size, ligand length, and environmental conditions on the MPI performance. Their findings revealed that chain formation in these nanoferrites occurred beyond a critical particle size and threshold field amplitude, enhancing their response under low drive field conditions. Figure 16 highlights the enhanced imaging resolution achieved by the authors, whereas figure 16(A) showcases a full-width at half-maximum (FWHM) analysis of the point-spread function (PSF) across different AC drive fields, demonstrating up to a 9× improved spatial resolution compared to commercial tracers. Figure 16(B) identifies the drive field threshold, where chain formation served as the dominant mechanism for signal enhancement, aligning with micromagnetic simulation results that indicated an energy crossover favoring particle chains. The sharpest PSF, shown in figure 16(C), confirmed the superior resolution of the materials over conventional tracers. Meanwhile, the trade-off between increased signal intensity and resolution sharpness emphasized the need for parameter optimization, as illustrated in figure 16(D).

The normalized signals in figure 16(E) show that 22 nm (sample S9) particles successfully resolved a phantom with a 2 mm separation, confirming their potential for high-contrast, high-precision biomedical imaging. In figure 16(F), a 2D MPI image is reconstructed using these nanoparticles, showcasing their ability to resolve a phantom with 2 mm spacing in both x and y directions. The image shows high contrast and precision, with signal strength varying according to nanoparticle concentration, as indicated by color mapping. These findings underscore the establishment of strongly interacting nanoparticles as promising candidates for *in vivo* imaging, tumor tracking, and thermometry applications.

#### 4.2.2. MPI for tumor imaging

**Figure 17.**
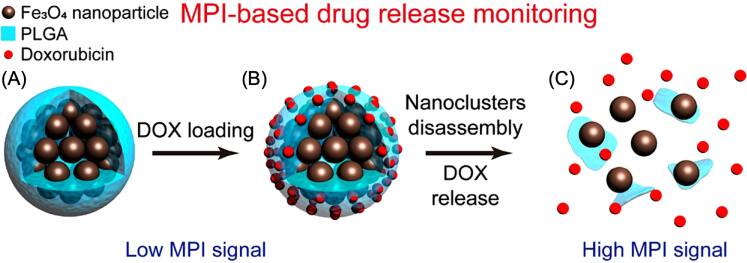
Schematic of a magnetic nanocomposite for MPI-based drug release monitoring. (A) core–shell structure of Fe_3_O_4_/PLGA nanocomposites with the magnetite core (brown) encapsulated in a PLGA shell (blue). (B) DOX (red) loaded on the nanocomposites for drug delivery and MPI-based release tracking. (C) In acidic environments, the PLGA shell degrades, releasing DOX and disassembling the Fe_3_O_4_ cores, leading to an increased MPI signal for drug release monitoring. Figure reprinted with permission from [224], copyright 2019 American Chemical Society.

As presented previously, MPI has emerged as a high-sensitivity, zero-background imaging modality with applications in tumor tracking, immune cell monitoring, drug delivery, and intraoperative imaging. Preclinical studies, such as those by Yu *et al*, have demonstrated its ability to monitor systemic accumulation of nanoparticles in tumors via passive EPR effects, achieving tumor-to-background ratios of >50 in rodent models [223]. MPI has also enabled the real-time monitoring of drug release dynamics in tumors, particularly through Fe_3_O_4_-based nanocomposites designed for controlled drug release and enhanced Brownian relaxation. For example, core/shell MNPs with a magnetite core and a polymer shell have been exploited for performing MPI-based drug release monitoring, as presented in figure 17 for a nanocomposite comprising Fe_3_O_4_ nanoparticles encapsulated within a PLGA shell and loaded with DOX. The release mechanisms of these materials involve the degradation of the PLGA shell in the tumor’s acidic environment, leading to the release of DOX, while achieving an enhancement of the MPI signal due to increased Brownian relaxation [224].

Additionally, this technology has been applied to longitudinal tumor imaging by tracking iron-labeled tumor cells from implantation to metastasis, with a positive correlation to histological findings [225]. Moreover, the ability of MPI to detect circulating tumor cells (CTCs) has been further demonstrated in tumor self-homing models, emphasizing its sensitivity in capturing CTC dynamics. For example, Makela *et al* applied MPI to the tracking of iron-labeled tumor cells *in vivo*, demonstrating the high sensitivity and quantitative potential of this technology [225]. In the study, MDA-MB-231BR breast cancer cells were labeled with micron-sized iron oxide particles and injected into mice harboring mammary fat pad tumors. Using MPI, they successfully detected the migration of iron-labeled CTCs to the primary tumor site 72 h post-injection, a phenomenon known as tumor self-homing. Furthermore, the authors validated their findings through *ex vivo* MPI, MRI, and histological analysis, confirming the retention of iron within metastatic cells.

**Figure 18.**
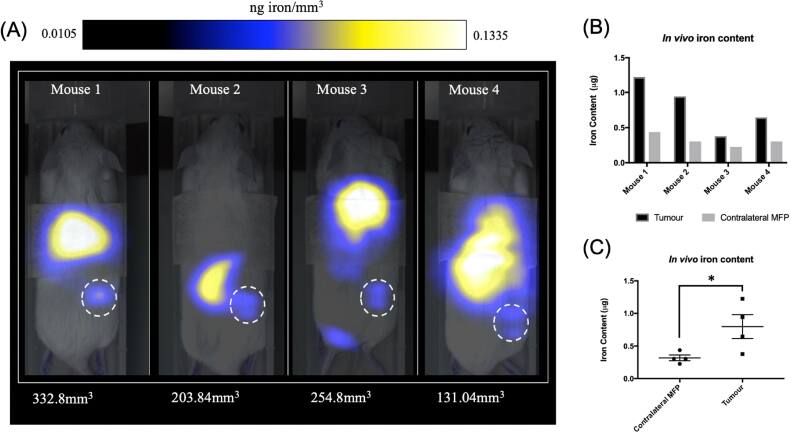
MPI’s capability for real-time tracking of iron-labeled CTCs. (A) Whole-body MPI scan shows hotspots of CTC accumulation at the tumor site, confirming tumor self-homing. (B) Iron content in tumor-bearing and contralateral mammary fat pads. (C) Quantification of iron retention, revealing a higher iron-labeled cell accumulation in tumors than in non-tumorous tissues. Figure reprinted with permission from [226], copyright 2021 The Royal Society of Chemistry.

In a similar work, Parkins *et al* explored the use of MPI for the longitudinal tracking of iron-labeled CTCs and their self-homing behavior [226]. In their study, MPI facilitated the real-time tracking of tumor cell dynamics, iron clearance mechanisms, and potential interactions between labeled and non-labeled cells. MDA-MB-231BR breast cancer cells were labeled with iron microparticles and injected into the left ventricle of tumor-bearing mice, mimicking a natural metastatic spread. MPI enabled highly sensitive detection and quantification of CTCs at the primary tumor site in the mammary fat pad, confirming their recruitment. Whole-body MPI scans (figure 18(A)) revealed a hotspot of CTC accumulation at the tumor site, confirming tumor self-homing. The relationship between CTC recruitment and tumor size was analyzed through tumor volume measurements by comparing the iron content present in the form of iron-labeled CTCs between the tumor-bearing mammary fat pad and the contralateral (non-tumorous) mammary fat pad (figure 18(B)), providing insight into metastatic progression. Quantification of iron retention (figure 18(C)) verified that iron-labeled cells accumulated more in tumors than in non-tumorous tissues.

**Figure 19.**
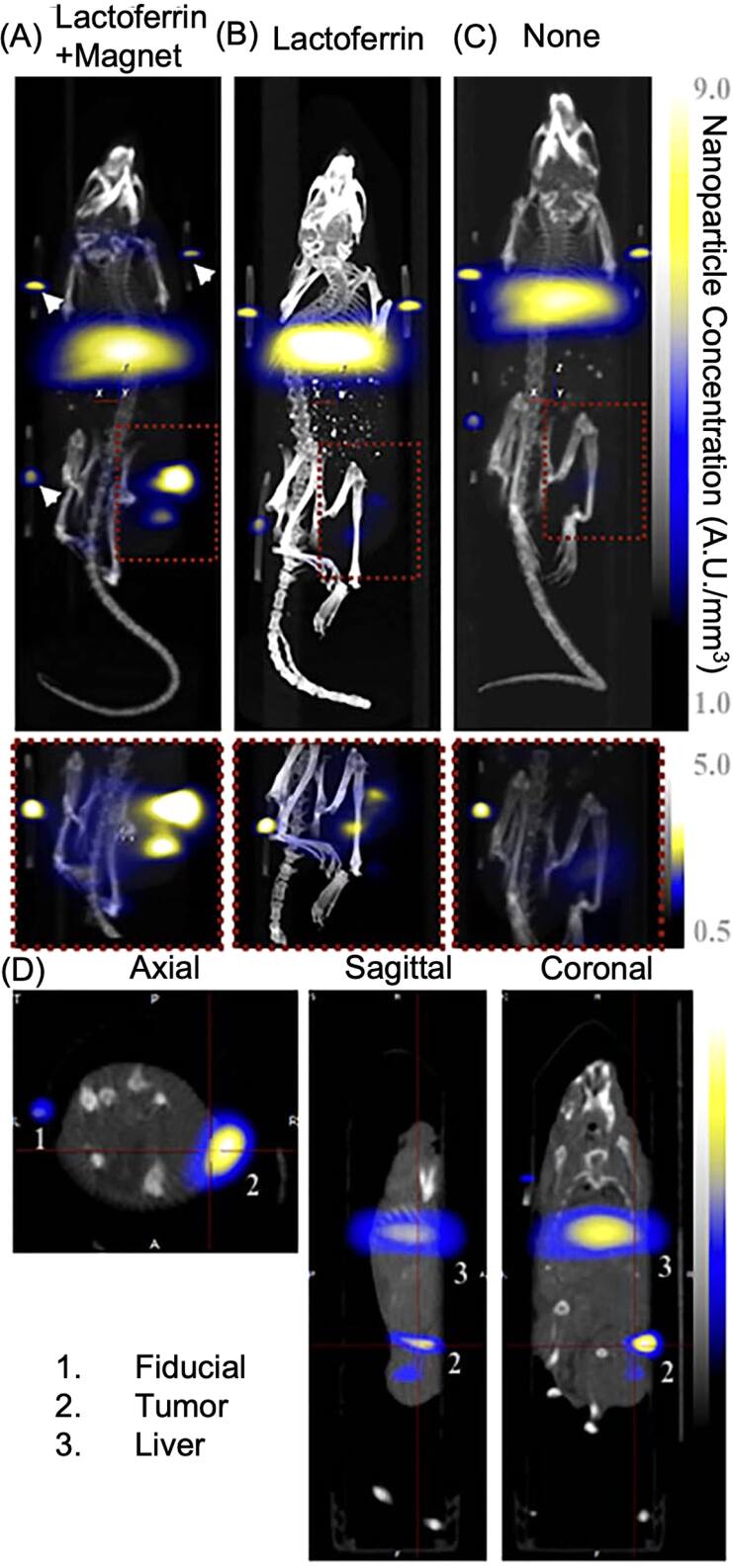
MPI images of tumor xenografts in mice, illustrating the enhanced targeting effect of lactoferrin-functionalized nanoparticles combined with magnetic field-assisted targeting. (A) MPI combined with CT (MPI/CT) overlay of mice injected with Cyanine5.5-lactoferrin conjugated MNPs and subjected to external magnetic targeting, showing enhanced tumor localization (red squares). (B) MPI/CT overlay of mice injected with lactoferrin-functionalized particles without magnetic targeting, demonstrating reduced material accumulation in the tumor. (C) MPI/CT overlay of mice injected with non-functionalized MNPs, highlighting the role of lactoferrin in facilitating tumor accumulation. (D) Axial, sagittal, and coronal views of the animal, confirming the 3D spatial distribution of MNPs in the tumor and liver. Figure reprinted with permission from [227], copyright 2017 The Royal Society of Chemistry.

In a different study, Arami *et al* employed MPI to track IONPs functionalized with lactoferrin for targeted brain cancer imaging, combining MPI with Computed Tomography (CT), demonstrating the MPI’s ability to provide a high-resolution, quantitative tomographic imaging [227]. By conjugating lactoferrin on the particle’s surface, a tumor-specific accumulation was observed, further improved by using an external magnet. Using MPI, these authors visualized only IONPs in tissues, eliminating the background interference from diamagnetic tissues, a key advantage over MRI. Their results showed high-contrast agent sensitivities, detecting 1.1 ng of iron with an SNR of ∼3.9, and achieving a spatial resolution of approximately 600 μm. The targeting efficiency of these lactoferrin-functionalized nanoparticles is presented in figure 19. Figures 19(A)–(C) present the imaging results of the tumor accumulation (red squares) of lactoferrin-functionalized particles with (figure 19(A)) and without an external magnetic field assistance (figure 19(B)), as well as non-functionalized particles (figure 19(C)). This indicates the importance of active targeting strategies using proper surface functionalization and magnetic field assistance. Multi-plane imaging, presented in figure 19(D), confirmed the 3D nanoparticle distribution within the tumor and clearance through the liver. These results demonstrate MPI as a highly sensitive and non-invasive imaging tool for cancer diagnostics, making it a powerful method to track tumor-targeting nanoparticles and refine nanomedicine therapies.

Advances in nanoparticle engineering have significantly enhanced MPI applications in cancer diagnostics and therapy. The case of Ozer *et al*’s work investigating the targeting effects of CoFe_2_O_4_ nanoparticles with biocompatible polyethyleneimine (PEI) coatings suggested selective toxicity of these materials towards glioma cells compared to healthy fibroblasts [228]. This selective targeting capability, combined with the magnetic properties of CoFe_2_O_4_ nanoparticles, indicates their potential for brain cancer (specifically glioblastoma) diagnosis and treatment. Moreover, immune cell tracking using MPI has enabled the non-invasive monitoring of chimeric antigen receptor (CAR)-T and CAR-Natural Killer (NK) cells, used for cancer immunotherapy, providing a radiation-free, highly sensitive tracking method [229]. Additionally, focused field-of-view techniques have been developed to isolate tumor signals from background liver signals, which is crucial for the accurate quantification of TAMs in breast cancer progression. This development was necessary because intravenously injected SPIONs used to label TAMs tend to accumulate in high concentrations in the liver, resulting in a high MPI signal from liver tissues, which masks regions of interest with lower signals (i.e., breast tumors), preventing their isolation and quantification. Thus, enabling focus on a smaller field of view around the tumor site has allowed accurate measurements of TAM presence in breast tumors without interference from the strong liver signals [230].

Furthermore, innovations in dual-modality imaging have further enhanced MPI’s diagnostic applications, such as the combination of MPI with fluorescence molecular imaging, which has improved breast cancer lymph node metastasis detection through deep tissue imaging and high-resolution fluorescence mapping [231]. In addition, intraoperative applications of MPI have been explored for tumor margin analysis during breast-conserving surgery, employing SPION tags for precise identification of residual tumorous tissue [232]. Efforts to enhance MPI performance have also focused on magnetic labeling optimization. Despite its suboptimal MPI properties, Resovist, a former MRI contrast agent, has become the benchmark MPI tracer due to its bimodal size distribution, which enhances imaging capabilities [233]. Tracer design refinements have emphasized that only 3% of Resovist’s particles contribute significantly to the generated MPI signal, indicating the need for optimized nanoparticle formulations [234]. Some studies have confirmed that SPIONs with large core sizes near the ferromagnetic transition range (20–30 nm) improve resolution, reinforcing the importance of a proper design of the nanoparticle size and surface coatings for enhancing MPI sensitivity [235]. Collectively, these advancements highlight ongoing developments in MPI for cancer imaging, immune cell monitoring, and therapy guidance, with a strong emphasis on tailored nanoparticle tracers or labels for improved imaging performance.

### 4.3. Biosensing and molecular diagnostics

#### 4.3.1. GMR-based biosensors for cancer diagnostics

GMR biosensors are based on spin-dependent electron scattering in multilayered structures composed of ferromagnetic and non-magnetic conductive layers [236, 237]. In the absence of an external magnetic field, the magnetizations of adjacent ferromagnetic layers are typically antiparallel, resulting in a high electrical resistance. When an external magnetic field is applied, the magnetizations get realigned into a parallel configuration, thereby reducing resistance [238, 239]. This resistance change is the basis for magnetic field detection in GMR sensors. In biosensing applications, GMR sensors are used for quantitative detection of biomolecules that are labeled with MNPs. The sensor surface is first functionalized with capture probes specific to target analytes, such as cancer biomarkers (e.g., proteins, circulating tumor cells, etc). Once the target binds to the capture probe, a secondary MNP-conjugated detection agent is introduced, forming a sandwich complex. The bound MNPs generate stray magnetic fields that perturb the magnetization state of the GMR layers, resulting in a quantifiable change in resistance. This electrical signal quantitatively correlates with the concentration of the biomarker, enabling a highly sensitive, label-based magnetic detection. In cancer diagnostics, GMR biosensors offer a rapid, quantitative detection of specific biomarkers at low concentrations, supporting early diagnosis and real-time monitoring.

GMR biosensors provide high sensitivity, low background noise, and multiplexing capabilities, making them suitable for the early detection of cancer biomarkers in point-of-care settings through the integration of multiple sensors on a single chip [27, 240]. Indeed, Gao *et al* employed a GMR-based immunoassay that simultaneously detected twelve tumor markers; such a device could enhance the diagnostic sensitivity and accuracy for detecting a variety of tumors, such as lung, liver, and prostate cancers [241]. Another study reported the development of a multiplexed GMR biosensor capable of detecting several ovarian cancer biomarkers, including Interleukin 6 (IL6), Cancer Antigen 125 (CA-125), and Human Epididymis Protein 4 (HE4), with high sensitivity and low detection limits, highlighting the potential of this technology for early cancer diagnosis in portable formats [32]. Additionally, Sun *et al* developed a GMR-based biosensor for the detection of carcinoembryonic antigen (CEA), a tumor marker for cancers such as colon and gastric tumors, with a detection limit as low as 10 pg ml^−1^ [33]. GMR biosensors have also been able to detect nucleic acids with high precision. For example, a recent study brings to light the detection of circulating tumor DNA fragments (ctDNA) using GMR biosensors, offering a low-cost and automated alternative for liquid biopsy applications in cancer diagnostics [242].

**Figure 20.**
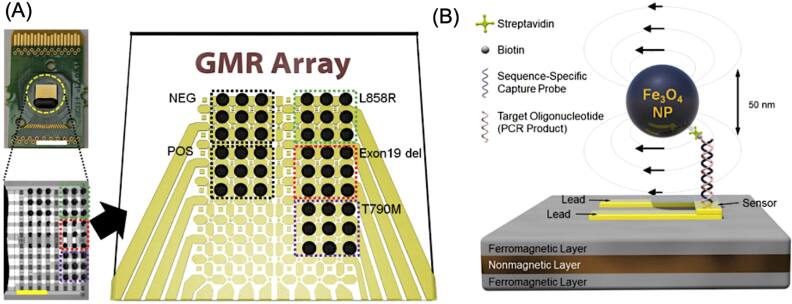
(A) Image of the GMR sensor chip (scale bar: 5 mm) with a magnified view of the 8 × 10 sensor array (scale bar: 0.5 μm). Each mutation-specific probe is replicated across 9 sensors for redundancy. (B) Schematic cross-section of the GMR sensor structure, illustrating MNP binding via biotin–streptavidin interactions to hybridized PCR products. The alternating ferromagnetic and non-magnetic conductive layers enable spin-dependent electron scattering, with MNPs inducing localized magnetic field changes that alter sensor resistance. Figure reprinted with permission from [243], copyright 2021 American Association for Clinical Chemistry.

Nesvet *et al* reported GMR biosensors (figure 20(A)) for detecting epidermal growth factor receptor (EGFR) gene mutations in ctDNA [243]. After the ctDNA was amplified using Polymerase Chain Reaction (PCR), biotin-labeled DNA fragments were attached to specific probes on the surface of the GMR sensor. Streptavidin-functionalized MNPs were attached to the biotin-labeled DNA through strong streptavidin–biotin interactions (figure 20(B)), and their presence generated localized magnetic fields that altered the sensor’s resistance in direct proportion to the amount of target DNA present. This MNP-mediated signal amplification enabled ultrasensitive detection of EGFR mutations at 0.01% mutant allelic fraction (equivalent to 3 pg mutant DNA in 30 ng total cfDNA), outperforming fluorescence-based methods by 250–1500× in sensitivity. MNPs also minimized background interference from non-target biomolecules, achieving a 96.3%–100% specificity. Furthermore, MNPs facilitated rapid (∼15 min) real-time measurements in a portable GMR system, allowing frequent therapy monitoring. The study validated this approach in 36 plasma samples, demonstrating 100% concordance between MNP-GMR results and radiographic outcomes for tyrosine kinase inhibitor (TKI) response prediction. MNPs’ stability and magnetic properties were pivotal in maintaining assay reproducibility and enabling point-of-care applications.

**Figure 21.**
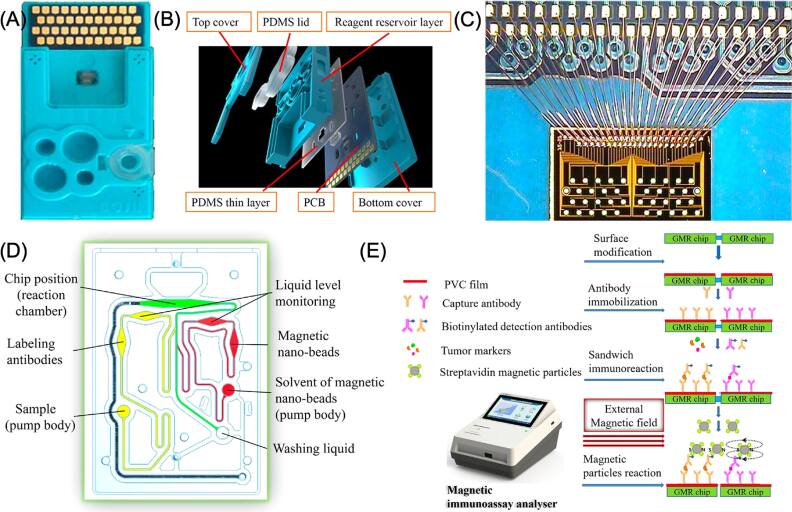
Structural and functional components of the GMR sensor for multi-biomarker immunoassay. (A) The test card used in the experiment. (B) The layered structure of the test card. (C) The GMR chip and its interface with the printed circuit board for connectivity. (D) The design of the microchannel system. (E) The reaction mechanism involved in the GMR multi-biomarker immunoassay. Figure reprinted with permission from [241], copyright 2018 Elsevier B.V.

In addition to their excellent sensitivity and high portability, GMR biosensors have demonstrated the ability to perform multiplexing analysis with good resolution. As mentioned previously, Gao *et al* developed a GMR multi-biomarker immunoassay for simultaneously detecting 12 tumor markers [241]. They integrated a GMR sensor chip, a microfluidic device, and magnetic nanobead labels into a point-of-care diagnostic system. The GMR chip featured 40 individual sensors, each with a diameter of 120 μm, enabling the simultaneous detection of multiple proteins from the sample. To enhance antibody immobilization, the researchers optimized the surface functionalization of the GMR sensors using Polyvinyl Chloride (PVC) coatings and UV-ozone treatments. They also designed an advanced microfluidic system with multiple channels to handle the sample, washing solution, and MNPs, facilitating automated and efficient immunoassay procedures. Figures 21(A)–(D) present the overall test card design, its various layers, the GMR chip connection to a microcontroller board, and the microchannel system. The biochemical reaction process and post-reaction detection of magnetic nanobeads are outlined in figure 21(E). A key innovation was their systematic evaluation of different biotin compounds for labeling detection antibodies, which was crucial for the biotin-avidin amplification system used to link antibodies to streptavidin-coated MNPs. They tested several biotin derivatives: NHS-Biotin, NHS-LC-Biotin, NHS-LC-LC-Biotin (amine-reactive, with increasing spacer arm lengths), Maleimide-Polyethylene Glycol (PEG2)-Biotin (sulfhydryl-reactive), and Hydrazide-LC-Biotin (carbonyl-reactive), to determine how reactive group and spacer length affect assay sensitivity and linear detection range. For certain markers (e.g., alpha-fetoprotein (AFP)), long spacer arms improved reactivity, likely by enhancing accessibility to buried binding sites, while for others, spacer length had little effect. Amine-reactive biotins generally showed the highest reactivity, and Hydrazide-LC-Biotin was found to be too weak for practical use. This optimization of biotin labeling chemistry contributed to the assay’s high sensitivity, wide dynamic range, and rapid detection of multiple cancer biomarkers in a clinical setting. The system achieved rapid detection, completing the analysis of all 12 tumor markers in just 15 min. This innovative GMR-based platform exhibited high sensitivity, a wide detection range, and excellent precision, positioning it as a promising tool for clinical cancer diagnostics.

#### 4.3.2. MTJ-based biosensors for cancer diagnostics

Magnetic tunnel junctions (MTJs) are spintronic devices comprising two ferromagnetic layers separated by an ultrathin insulating barrier, typically magnesium oxide (MgO). The electrical resistance of an MTJ is directly dependent on the relative magnetic orientation of these two ferromagnetic layers. A parallel alignment of magnetization results in a low resistance, whereas an antiparallel alignment leads to a high resistance [244, 245]. This phenomenon, known as the tunneling magnetoresistance (TMR) effect, enables MTJs to function as highly sensitive magnetic field sensors. For biosensing applications, MTJs are commonly employed in a spin-valve configuration. In this architecture, one ferromagnetic layer, referred to as the pinned layer, possesses a fixed magnetization orientation. The magnetization direction of the second ferromagnetic layer, the free layer, is designed to rotate in response to an external magnetic field [246]. In quantitative bioassay applications, MNPs are immobilized onto the free layer of the MTJ via a sandwich assay mechanism, as detailed in section 4.3.1. When an external alternating current (AC) magnetic field is applied, these MNPs perturb the magnetization of the free layer. This perturbation, in turn, induces a measurable change in the MTJ’s resistance, providing a quantitative means to detect and estimate the concentration of target analytes. In cancer diagnosis, MTJs are employed to detect MNPs functionalized to bind to specific cancer biomarkers. For instance, MTJ sensors have been used to detect proteins such as AFP, which can be used to identify the presence of tumors when labeled with MNPs [244]. Indeed, in the investigation carried out by Lei *et al,* the authors showcased the use of an MgO-based MTJ sensor for detecting AFP, an important biomarker for liver cancer. The researchers developed a magnetic immunoassay using MTJ sensors with a sensing area of 4 × 2 μm^2^ and 20 nm iron-oxide MNPs as labels to detect AFP in a sandwich-assay configuration. The MTJ sensors exhibited a high TMR of 122% and a sensitivity of 0.95%/Oe at room temperature. The system successfully detected AFP at concentrations as low as 0.002 mg ml^−1^, with the sensor’s resistance changing logarithmically as a function of the AFP concentration. This work showcases the feasibility of MTJ-based biosensors for early-stage liver cancer detection, offering advantages such as high sensitivity, low cost, and portability over conventional immunoassay techniques.

The high sensitivity of MTJ sensors enables the detection of very small quantities of MNP labels, offering the potential for early-stage cancer diagnosis. Compared to conventional methods like fluorescence immunoassays, MTJ-based biosensors offer advantages including greater sensitivity, reduced cost, and the potential for miniaturization and integration into lab-on-a-chip platforms. For example, Cousins *et al* demonstrated a novel handheld magnetometer probe based on MTJ sensors for intraoperative sentinel lymph node identification [245]. This probe employed highly sensitive MTJ sensors to detect MNPs that had been previously accumulated in sentinel lymph nodes. The researchers achieved an excellent spatial resolution of 4.0 mm and demonstrated the ability to detect as little as 5 μg of MNPs. In preclinical animal experiments, the probe successfully identified all first-tier nodes with no false positives or negatives. The high sensitivity and spatial resolution of this MTJ-based probe make it particularly promising for use in complex lymphatic environments, such as those encountered in gastrointestinal cancers. This innovation addressed the limitations of conventional gamma probes by providing improved detection capabilities without relying on radioactive materials, representing a significant advancement in MNP detection for cancer staging and treatment planning.

#### 4.3.3. MPS-based biosensors for cancer diagnostics

**Figure 22.**
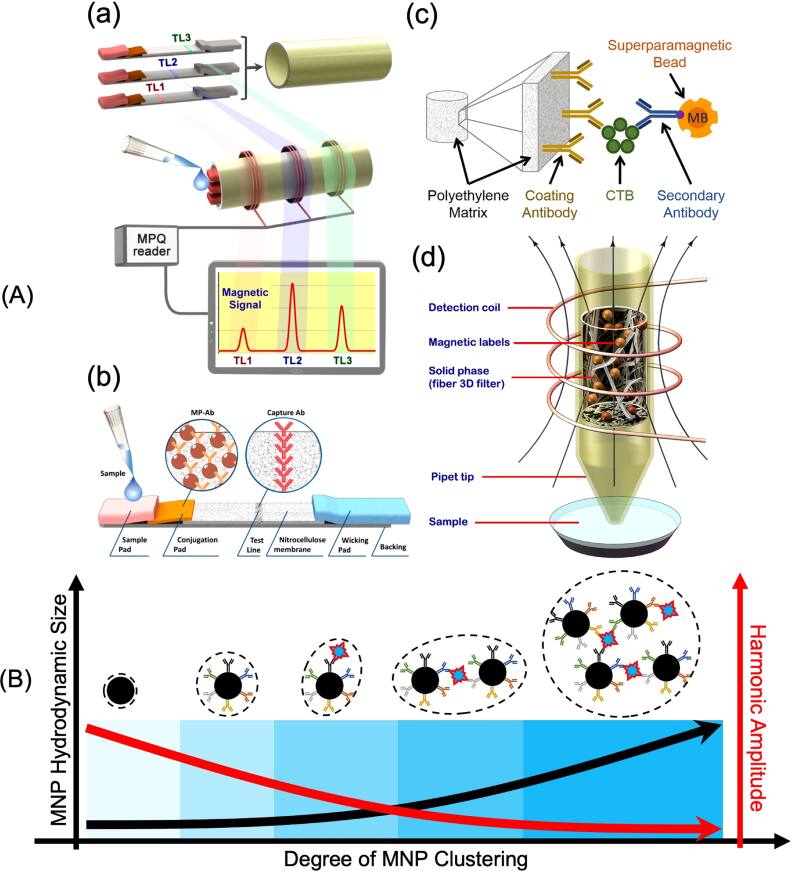
(A) Surface-based MPS bioassay workflow. (a) Multiple test strips are assembled into a compact cartridge (top). After depositing the sample onto the cartridge’s front end, the cartridge is inserted into a portable MPS reader (middle), enabling simultaneous detection of magnetic signals from all strips (bottom). (b) One strip configuration employs a sandwich lateral flow (LF) format, where antibody-functionalized MNPs serve as detection labels. (c) Illustration of a magnetic sandwich immune-filtration assay implemented with a 3D fiber filter. (d) Diagram showing a single 3D solid-phase fiber filter housed within a pipette tip. (B) Volumetric-based MPS bioassay principle. Upon binding to target analytes, MNPs form larger clusters via interactions with other particles in solution, increasing their hydrodynamic diameter. This aggregation reduces their dynamic magnetic response, observed as a decrease in harmonic amplitude. (A:a) and (A:b) reprinted with permission from [255], copyright 2016 American Chemical Society. (A:c) reprinted from [256], licensed under CC BY 4.0. (A:d) reprinted with permission from [257], copyright 2012 American Chemical Society. (B) reprinted with permission from [258], copyright 2020 American Chemical Society.

MPS is a technique derived from MPI that utilizes the nonlinear magnetization response of MNPs to an external AMF. In the presence of AC magnetic fields, the non-linear response of MNPs results in higher harmonic components in their magnetization response [247, 248]. These higher harmonics are highly sensitive to the hydrodynamic size of the particles [249], and the temperature and viscosity of the surrounding media [250–253], enabling the precise detection of analytes in complex biological environments. Utilization of superparamagnetic MNPs also offers the advantage of a high contrast, as the biological samples are largely non-magnetic or diamagnetic. This capability makes MPS particularly valuable for bioassays and cancer diagnostics, as it can identify molecular interactions, such as the binding of MNP-labeled probes to tumor-specific antigens. MPS can be employed in two primary ways for bioassay purposes: surface-based and volumetric-based bioassays. In surface-based MPS, a functionalized substrate captures MNPs in the presence of specific biomarkers, forming a sandwich complex, where the MPS harmonic signal is directly proportional to the number of bound MNPs, and thus, quantitatively reflects the presence of target analytes in the test media. Figure 22(A) showcases a surface-based MPS bioassay, where functionalized substrates serve to immobilize MNPs using a sandwich assay mechanism. The setup can incorporate lateral flow (LF) strips (figures 22(A: a and b)) and 3D fiber filters (figures 22(A: c and d)), enabling target analyte binding and retention. After a washout step to remove unbound MNPs, MPS signals are read, allowing for the detection of retained MNPs. In contrast, volumetric-based MPS detects biomarkers in a liquid phase, where binding events alter the dynamic relaxations of MNPs, thereby affecting their collective magnetization response. In volumetric-based MPS bioassays, MNPs coated with polyclonal antibodies are used, which form clusters upon interacting with target analytes. This increase in hydrodynamic size impacts their dynamic magnetization response, manifesting as a corresponding reduction in the MPS harmonic spectra, as depicted in figure 22(B) [35, 37]. Both surface-based and volumetric-based MPS methods enable low-cost, highly sensitive, and quantitative detection of a wide range of biomarkers, with surface-based implementations also offering the potential for multiplexed bioassays [254].. MPS offers several key advantages over traditional analyte detection methodologies, including high sensitivity, rapid testing, and the capability for point-of-care diagnostics, positioning it as a powerful tool for cancer detection and monitoring.

Within cancer applications, for instance, Wei *et al* reported carboxyl-coated multi-core MNPs Synomag^®^-30 for cancer cell detection using MPS [35]. These multi-core MNPs were approximately 30 nm in size, and their coating facilitated efficient uptake into human neuroblastoma cells (the SK-N-SH cell line was used in this study). When SK-N-SH cancer cells were incubated with these MNPs, each cell took up about 64 pg of iron and stayed healthy. They found that when these MNPs aggregated inside the cells, the MPS signal changed, enabling easier quantification of cell numbers. By measuring the third harmonics, they could detect as few as 200 labeled cells in a sample. This study highlighted that both the design of the MNPs and how they behave inside cells are very important factors for making MPS a powerful tool for tracking cancer cells during therapy.

#### 4.3.4. NMR-based biosensors for cancer diagnostics

NMR leverages the magnetic properties of certain nuclei, such as hydrogen, that have an odd number of protons or neutrons. When placed in an external magnetic field, these nuclei align either in parallel (low energy) or antiparallel (high energy) to the field, behaving like tiny magnets. An RF pulse at the resonance frequency excites the nuclei from the low-energy to the high-energy state. Once the RF pulse is turned off, the nuclei relax back to their original alignment, emitting RF signals known as free induction decay (FID), which are detected by the NMR system. Fourier transformation of the detected signal converts it into a frequency spectrum, providing information about the chemical environment of the nuclei and revealing molecular structures and compound concentrations.

**Figure 23.**
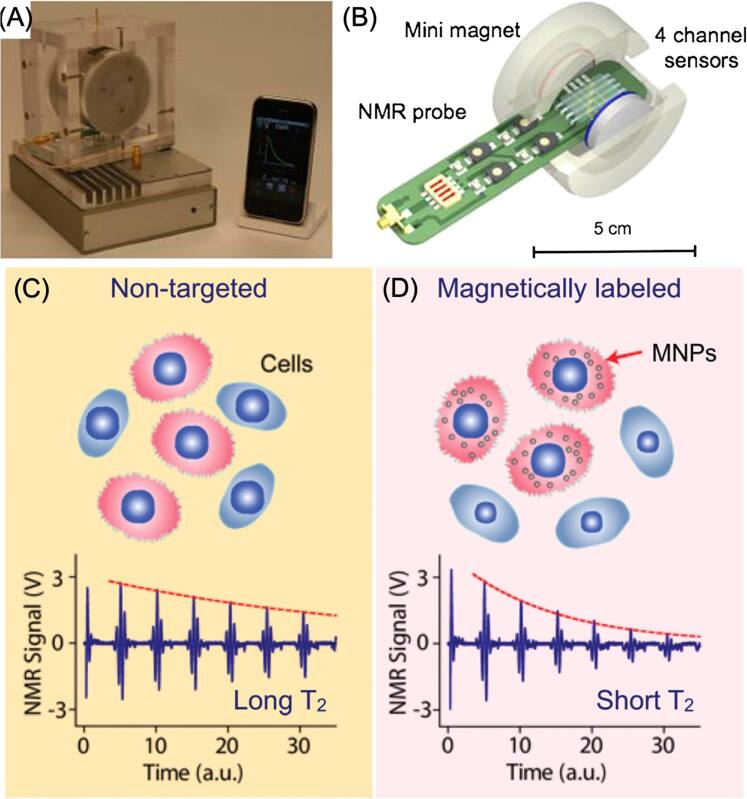
(A) μNMR system for point-of-care diagnosis. The lower compartment consists of the necessary components for NMR measurements, whereas the top compartment houses a permanent magnet with chip-sized volume sensors. (B) Internal assembly of the μNMR probe used for sensing within the mini magnet. (C) Time-domain signals showing slower decay for control cells versus MNP-tagged cells (D), indicating increased relaxation rates under MNP presence. (A) and (B) reprinted with permission from [260], copyright 2014 Springer Science Business Media New York. (C) and (D) reprinted with permission from [39], copyright 2014 The Royal Society of Chemistry.

Recent advancements in NMR technology have been utilized for biosensing applications, particularly in tumor diagnostics. For instance, in the study reported by Haun *et al* [259], the authors demonstrated the use of micro-NMR technology (μNMR) for the multiplexed analysis of tumor cells obtained via fine-needle aspirates, achieving a 96% diagnostic accuracy by quantifying protein markers using MNPs to enhance NMR signals. The compact system (10 cm × 10 cm) integrated microfluidics for handling small cell samples, enabling a rapid and multiplexed molecular analysis of human tumors. The fully integrated μNMR system was developed for bedside clinical diagnostics, as seen in figure 23(A), and included a compact unit housing circuitry for NMR measurements, along with a built-in permanent magnet and microliter-volume sensors. The device design incorporated an advanced μNMR probe (figure 23(B)), optimized for high-sensitivity detection within the miniaturized magnetic setup, to ensure accurate and efficient clinical assessments. The device proved to be able to overcome the limitations of conventional tumor diagnostics, such as long processing times, insufficient sensitivity to detect protein markers in small or degraded tumor samples, and the lack of real-time, quantitative analysis at the point of care. By leveraging MNPs and NMR-based detection, the platform enabled rapid molecular profiling. Tumor cells were labeled with MNPs conjugated to antibodies targeting specific protein markers, with each MNP creating a localized magnetic field that affected the surrounding water molecules’ spin dynamics. The μNMR platform measured the T_2_ relaxation times of water protons near the MNP-labeled cells. When MNPs bound to their target proteins on tumor cells, they caused a pronounced T_2_-shortening effect, increasing the decay rate of the NMR signal. This system quantified multiple protein markers simultaneously, such as HER2, EGFR, Mucin 1 (MUC-1), and tumor protein P53 (p53), using different MNP probes, enabling the identification of unique molecular signatures of cancer cells. The μNMR device demonstrated high sensitivity, detecting protein markers in samples containing as few as 200 cells. By analyzing a four-protein signature, the platform achieved a diagnostic accuracy of 96%, outperforming conventional immunohistochemistry. Moreover, the results were obtained within 60 min, making it ideal for clinical applications. The system also revealed significant variability in protein expression across different tumor samples and even within the same tumor, underscoring the importance of personalized diagnostics. Furthermore, automated analysis simplified the diagnostic process, making it suitable for point-of-care use.

In a different study, Castro *et al* developed a μNMR system to detect and profile CTCs by labeling them with immunospecific MNPs, leveraging the T_2_ relaxation effect to enhance sensitivity and minimize cell loss during liquid biopsy analysis [39]. The μNMR system achieved a high sensitivity by engineering MNPs, obtaining enhanced transverse relaxivity, r_2_, as seen in figures 23(C) and (D) in comparison to the control target cell group having no MNP tags. This was accomplished through the development of manganese-doped magnetite nanoparticles with sizes up to 22 nm using seed-mediated growth, as well as the creation of novel MNPs (Fe@MnFe_2_O_4_) featuring a hybrid structure with an elemental Fe core and a protective ferrite shell. These advancements led to a >400% increase in r_2_ compared to magnetite-based MNPs. To further improve detection, the study utilized tetrazine (Tz) and trans-cyclooctene (TCO) chemistry for an efficient MNP labeling of cells. A two-step approach was employed, using TCO-modified antibodies and Tz-loaded MNPs, which resulted in ∼15-fold improvements in MNP loading on cells compared to direct antibody-MNP conjugates. Sensitivity was further amplified by modifying secondary Immunoglobulin G (IgG) antibodies with TCO, boosting signal intensity and detection capability. Multiple prototypes of μNMR probes were explored. The first version (μNMR-1) demonstrated the feasibility of system miniaturization, reducing sample volumes to increase analyte concentrations and improve detection limits. Further refinements led to the creation of smaller NMR probes (coils) capable of generating stronger RF magnetic fields per unit current, significantly enhancing sensitivity. This work aimed to overcome technical challenges in current CTC research, enabling rapid and highly sensitive biomarker detection.

Finally, a few recent studies reported the high resolution of NMR-based analysis in cancer research. For instance, Beckonert *et al* employed high-resolution 1H-NMR spectroscopy to analyze metabolic changes in breast cancer tissues, identifying elevated levels of taurine and choline-containing compounds as malignancy markers [261]. A novel approach by Perumal *et al* introduced 19F NMR ON/OFF nanoparticles to detect cancer biomarkers, where MNP accumulation and disassembly altered T_2_ relaxation, enabling specific biomarker identification [262]. Last, and also using 1H-NMR, Wang *et al* identified a biomarker panel for hepatocellular carcinoma progression, including taurine and putrescine, and highlighted their roles in disease-associated metabolic pathways [263].

## 5. Challenges and future directions

MNPs hold immense potential for theranostic cancer treatment, as they offer integrated solutions for diagnosis, therapy, and real-time monitoring. However, several critical challenges must be overcome before these technologies can achieve widespread clinical adoption and regulatory approval. One major concern lies in the long-term toxicity and biocompatibility of MNPs, particularly IONPs [264–268]. While iron oxide is generally considered less toxic than other metals such as copper or cadmium, the large doses required for therapeutic efficacy raise questions about cumulative toxicity, iron overload, and potential oxidative stress in biological systems. The metabolic fate, biodegradation rate, and clearance pathways of MNPs must be thoroughly understood through long-term *in vivo* studies.

Another challenge comes from the reproducible synthesis and optimization of MNPs. Properties such as size, shape, crystallinity, surface charge, and magnetic behavior critically influence their therapeutic and diagnostic performance. Yet, due to the inherent variability in nanoscale synthesis processes, achieving batch-to-batch consistency remains difficult. Even within a single batch, polydispersity in size and magnetic response can lead to unpredictable outcomes. Standardized protocols and quality control measures are urgently needed to ensure uniformity and reproducibility.

Additionally, modeling the dynamic behavior of MNPs under physiological conditions and in complex magnetic fields poses computational challenges. Accurate simulation of MNP magnetization, relaxation dynamics, and *in vivo* transport often requires high-fidelity, resource-intensive models. These simulations are essential for optimizing particle design and predicting interactions under various clinical scenarios, yet they remain inaccessible to many research groups due to computational cost and model complexity.

There are also practical and regulatory challenges. Scalable manufacturing of MNPs with consistent quality is not yet routine, and current surface functionalization (i.e., coatings) strategies may face difficulties during upscaling or storage. These issues may not satisfy established good manufacturing practices (GMP), which would make it difficult to commercialize MNPs and thus implement them into clinical settings at a large scale [269]. Furthermore, regulatory pathways for theranostic agents that serve both diagnostic and therapeutic functions are more complex than for conventional drugs or imaging agents. Regulatory approval will depend on a robust demonstration of safety, efficacy, and reproducibility across preclinical and clinical studies.

Further, the efficacy of MNP treatments for humans remains under question, as rodents are the main test subject for most animal-based MNP research. These animals are smaller mammals and have less depth required for AMFs to effectively reach and treat tumors utilizing hyperthermia with minimal to no side effects. It may be more challenging to penetrate deeply into larger specimens, such as big mammals or humans, without eddy currents causing excessive electromagnetic exposure [270]. Hyperthermia becomes more complex if the AMF cannot reach the injected MNPs at a desired intensity without causing undue harm. Rodents and humans differ in physiological ways that make translation from murine research to clinical trials difficult. Another consideration, including these differences, is the enhanced permeation and retention (EPR) effect of tumors. Murine xenograft tumor models tend towards accumulating and retaining more MNPs in comparison to human tumors [271], where the EPR is generally weaker, and strong heterogeneity is present. As a result, existing MNP theranostic research *in vivo* may overestimate the uptake of MNPs into tumors when considering only studies using mice.

Despite these challenges, the future of MNP-based theranostics is promising. Technological advances in synthesis, surface engineering, imaging hardware, and computational modeling continue to improve the precision and versatility of MNP systems. Innovations such as cell membrane coatings, responsive surface chemistries, and multifunctional hybrid nanoparticles are expanding the capabilities of MNPs for targeted, minimally invasive cancer treatment. Continued interdisciplinary research and collaboration across nanotechnology, materials science, oncology, and regulatory science will be essential to translate these advances into clinical reality, and a deeper understanding of targeting specificity will play an increasingly central role in that progress. Achieving high selectivity for cancer cells while minimizing uptake in healthy tissues remains a key requirement for clinical translation. Although passive targeting via the EPR effect and biologically inspired coatings such as cell membrane camouflage can improve circulation and tumor tropism, they still allow substantial accumulation in the liver and spleen [272–274]. Ligand-based active targeting provides receptor-specific recognition and internalization, offering greater tumor selectivity than passive approaches; however, its performance depends on heterogeneous receptor expression across tumor types. For this reason, the most promising strategies will likely combine biomimetic coatings and ligand-mediated targeting with active magnetic guidance, enabling both biochemical and physical control of MNP localization *in vivo*.

## 6. Conclusions

This review has provided a comprehensive overview of the fundamental properties, types, and biomedical applications of MNPs, with a focus on their emerging role in cancer theranostics. We began by examining the physical and magnetic characteristics of MNPs that underpin their suitability for biomedical use, followed by a discussion of surface functionalization strategies that enhance biocompatibility, circulation time, and targeting capability. The theranostic applications of MNPs, including magnetic hyperthermia, targeted drug and gene delivery, MRI, MPI, and biosensing, were reviewed, along with recent research findings and clinical developments. Finally, key challenges and future directions for advancing MNP-based cancer theranostics were explored.

MNPs offer distinct advantages over conventional platforms due to their controllable magnetic behavior, high biocompatibility, and multifunctionality. Their ability to be manipulated and directed using external magnetic fields enables precise spatial control for targeted drug delivery and minimally invasive therapy. Unlike ionizing radiation-based techniques, MRI and MPI utilizing MNPs are safer alternatives for imaging. Furthermore, MNPs can serve multiple roles, simultaneously acting as imaging contrast agents, therapeutic delivery vehicles, and hyperthermia agents, making them highly versatile tools in cancer treatment. Their tunable surface chemistry also enables the design of personalized therapies tailored to specific tumor types or patient needs.

Despite these promising features, several challenges need to be addressed before MNP-based theranostics can become a clinical standard. Key issues include concerns over long-term toxicity, variability in targeting efficiency, lack of standardized clinical protocols, and difficulty in achieving consistent, scalable nanoparticle synthesis. Addressing these limitations will require extensive research into long-term *in vivo* effects, advanced surface coatings tailored for complex biological environments, and the development of reproducible and scalable fabrication techniques. Additionally, more realistic and computationally efficient modeling approaches must be developed to simulate MNP behavior under physiological and clinical conditions.

Looking ahead, while the field of MNP-based cancer theranostics is still evolving, continuous advances in materials science, surface engineering, imaging technologies, and computational modeling continue to push the boundaries of what is possible. With sustained interdisciplinary efforts, MNPs have the potential to become a cornerstone of personalized, non-invasive, and highly effective cancer diagnosis and treatment.

## Data Availability

No new data were created or analysed in this study.
